# Perturbation of intestinal stem cell homeostasis and radiation enteritis recovery via dietary titanium dioxide nanoparticles

**DOI:** 10.1111/cpr.13427

**Published:** 2023-02-16

**Authors:** Linpei Zhang, Yinli He, Lele Dong, Chang Liu, Lin Su, Ruirui Guo, Qinying Luo, Baoyu Gan, Fang Cao, Yawen Wang, Haiyun Song, Xiaojiao Li

**Affiliations:** ^1^ BioBank The First Affiliated Hospital of Xi'an Jiaotong University Xi'an Shaanxi China; ^2^ Department of Pharmacy The First Affiliated Hospital of Xi'an Jiaotong University Xi'an Shaanxi China; ^3^ School of Public Health Shanghai Jiao Tong University School of Medicine Shanghai China; ^4^ Center for Translational Medicine The First Affiliated Hospital of Xi'an Jiaotong University Xi'an Shaanxi China

## Abstract

Small intestinal health and enteritis incidence are tightly coupled to the homeostasis of intestinal stem cells (ISCs), which are sensitive to dietary alterations. However, little is known about the impact of food additives on ISC pool. Here, we demonstrate that chronic exposure to low‐dose TiO_2_ NPs, a commonly used food additive, significantly hampers primary human and mouse ISC‐derived organoid formation and growth by specifically attenuating Wnt signal transduction. Mechanistically, TiO_2_ NPs alter the endocytic trafficking of the Wnt receptor LRP6 and prevent the nuclear entry of β‐catenin. Notably, dietary TiO_2_ NPs elicit modest chronic stress in healthy intestines and considerably impede the recovery of radiation enteritis by perturbing the homeostasis of ISCs in vivo. Our results identify a health concern of TiO_2_ NP exposure on ISC homeostasis and radiation enteritis recovery. These findings suggest extra precaution during the treatment of radiation enteritis and provide new insights into food additive‐ISC interaction.

## Graphical abstract

Titanium dioxide nanoparticles (TiO_2_ NPs), one of the most widely used food additives, propel the Wnt signalling receptor LRP6 from caveolin‐mediated endocytosis to clathrin‐mediated endocytosis to weaken the Wnt signalling activity, which is essential for the homeostasis of small intestinal stem cells (ISCs). Chronic intake of waterborne TiO_2_ NPs perturbs the homeostasis of ISCs and impedes the recovery of radiation enteritis.

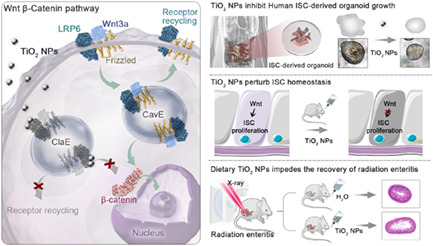

## 1 INTRODUCTION

The small intestinal epithelium is responsible for food digestion and nutrient absorption and plays a critical role in microorganism defence and immune response.^1^, ^2^ Since toxins in food can directly contact the intestine and induce intestinal epithelial cell (IEC) death, a rapid turnover of IECs is critical to maintain the integrity of small intestines.^1^, ^3^ Intestinal stem cells (ISCs), which are capable of both self‐renewal and differentiation into enterocytes or secretory‐lineage cells,^4^, ^5^ are indispensable for epithelial renewal. ISCs can derive single crypt to form intestinal organoid (also called mini‐guts) with 3D structure in vitro.^6^ Numerous studies have demonstrated that ISCs are closely related to the health and enteritis incidence of small intestines.^1^, ^7^, ^8^, ^9^ Recent evidence indicates that the self‐renewal and differentiation of ISCs can be controlled at least in part by dietary patterns including caloric restriction,^10^ fasting,^11^, ^12^ high‐fat diets,^13^, ^14^ ketogenic diets and high‐carbohydrate diets as well as other nutrients.^15^, ^16^, ^17^, ^18^, ^19^ However, little is known about the impact of food additive exposure on ISC homeostasis.

Nanoparticles (NPs) have brought a range of benefits to the food sector. Nanoscale titanium dioxide (TiO_2_) is one of the most widely manufactured NPs and accounts for more than 36% of TiO_2_ particles worldwide.^20^ Currently, as a food additive, TiO_2_ NPs have been added to more than 900 foods to enhance the opacity and brightness of products.^21^, ^22^ Candies, sweets and chewing gums have the highest content of TiO_2_ NPs.^20^ In addition, since TiO_2_ NPs can considerably prevent microbial growth and reduce *E. coli* contamination of food surfaces, they have been commonly used in food packaging and storage.^23^ Given that TiO_2_ NPs have been widely applied in the food industry, growing concerns about the potential health risks of TiO_2_ NPs on the intestines have been raised. Researchers found that foodborne TiO_2_ NPs could induce strong gut microbiota dysbiosis, colonic inflammation and proteome alterations in obese mice.^24^ Additionally, long‐term intake of the food additive TiO_2_ NPs altered the intestinal epithelial structure.^25^ Our previous study also revealed that dietary TiO_2_ NPs impeded the recovery of intestinal mucosal damage in colitic mice.^26^ Nevertheless, studies of food additive TiO_2_ NPs in ISC homeostasis are still lacking.

In clinic, radiation enteritis is inflammation of the intestines that occurs after radiation therapy, a primary treatment for malignant diseases and is commonly administered to patients with gynaecological, urological and gastrointestinal cancers. Almost all patients receiving pelvic or abdominal radiotherapy experience radiation enteritis,^27^ which usually manifests as pain, nausea, bloating and diarrhoea.^28^ Studies have demonstrated that ISCs are responsible for the recovery of radiation enteritis.^29^ However, whether chronic TiO_2_ NP intake influences the recovery of radiation enteritis remains poorly understood.

In this study, we investigated the impact of TiO_2_ NPs on the homeostasis of small intestinal stem cells. Our results showed that TiO_2_ NPs were mainly internalized via clathrin‐mediated endocytosis pathway during intestinal epithelial cell metabolism. Analysis of primary mouse and human organoids revealed that TiO_2_ NPs perturbed the homeostasis of ISCs by specifically attenuating Wnt signalling. At the molecular level, we demonstrated that TiO_2_ NPs triggered non‐canonical endocytic trafficking of the Wnt receptor low‐density lipoprotein receptor‐related protein 6 (LRP6) and prevented the nuclear entry of β‐catenin to weaken the outputs of Wnt signalling. Moreover, blockade of Wnt signalling by TiO_2_ NPs elicited modest chronic stress in healthy intestines and markedly impaired the recovery of radiation enteritis by inhibiting the proliferation of ISCs in both *Lgr5‐eGFP‐IRES‐creERT2* and C57BL/6 wild‐type (WT) mice. Together, our work revealed the impact of chronic TiO_2_ NP exposure on the homeostasis of ISCs and the recovery of radiation enteritis, and disclosed the mechanism underlying these effects. These findings give insights into the treatment of radiation enteritis, and expand our understanding of the food additive‐bio effects.

## 2 RESULTS

### 2.1 The cytocompatibility of TiO_2_ NPs

The physiochemical properties of TiO_2_ NPs used in our study have been characterized by transmission electron microscope (TEM) and dynamic light scattering (DLS) analyses.^26^ The uptake efficiency and mechanism of TiO_2_ NPs were explored in the mouse colon cancer cell line CT26. There are three main types of endocytosis in nonphagocytic cells: clathrin‐mediated endocytosis (CME), caveolin‐mediated endocytosis (CavMe) and micropinocytosis.^30^ Our TEM analysis demonstrated a rapid internalization of TiO_2_ NPs in CT26 cells, while the uptake of TiO_2_ NPs was significantly suppressed when cells were pre‐incubated with sucrose, genistein and cytochalasin D, which are known as inhibitors for CME, CavMe and macropinocytosis, respectively (Figure S1A). In particular, compared with other inhibitors, pre‐incubation of CT26 cells with sucrose dramatically impaired the cyto‐endocytosis of TiO_2_ NPs (Figure S1B), suggesting that TiO_2_ NPs were mainly internalized via the CME pathway. We then examined the cytotoxicity of TiO_2_ NPs in CT26 cells. The results showed that TiO_2_ NPs had a negligible influence on the viability of CT26 cells at concentrations between 10 μg/mL and 150 μg/mL within 48 h (Figure S1C,D). The results suggested a good cytocompatibility of TiO_2_ NPs in intestinal epithelial cells.

### 2.2 TiO_2_ NPs perturb ISC homeostasis in mouse intestinal organoids by specifically suppressing Wnt signalling

**FIGURE 1:**
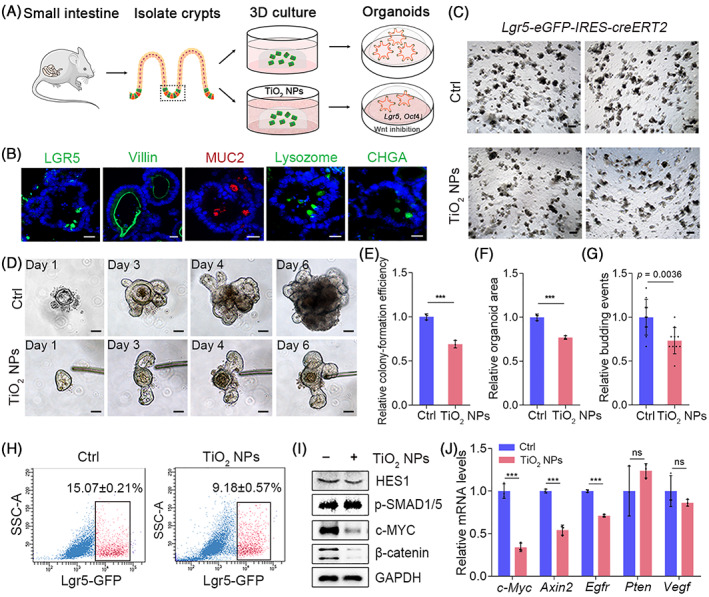
TiO_2_ NP exposure perturbs ISC homeostasis in mouse intestinal organoids by blocking Wnt signalling. (A) Schematic representation of the impact of TiO_2_ NPs on mouse intestinal organoids. (B) Morphology and composition of C57BL/6 WT mouse‐derived intestinal organoid. Scale bars, 20 μm. (C) Five days after culturing, TiO_2_ NP exposure hampered the formation and budding events of organoids derived from *Lgr5‐eGFP‐IRES‐creERT2* mice. Scale bars, 200 μm. (D) Time course of an isolated single crypt growth in the absence or presence of 50 μg/mL TiO_2_ NPs. Scale bars, 50 μm. (E, F) Colony‐forming efficiency (E) and size of mouse intestinal organoids (F) in each group. (*n* = 3, approximately 400 organoids per well were counted for calculation). (G) The budding events of mouse intestinal organoids under indicated conditions. (H) The impact of TiO_2_ NPs on Lgr5‐GFP^+^ ISC proportion was determined by FACS. (I) The effects of TiO_2_ NPs on the levels of the indicated proteins in the 3D organoids. (J) The transcriptional outputs of the indicated genes in the absence or presence of TiO_2_ NPs. Data are represented as the mean ± SD (*n* = 3). Student's *t*‐test, ns means not significant, ***p < 0.001.

Although incubation with a wide range of TiO_2_ NPs did not compromise the viability of intestinal epithelial cells, we continued to investigate whether uptake of TiO_2_ NPs could affect the homeostasis of ISCs in mouse intestinal organoids (Figure 1A). ISCs are characterized as Lgr5‐GFP^+^ cells and CD44^+^CD24^lo^CD166^+^GRP78^lo/−^ cells in *Lgr5‐eGFP‐IRES‐creERT2* mice and C57BL/6 WT mice, respectively.^31^, ^32^ Given that *Lgr5‐eGFP‐IRES‐creERT2* mice can directly identify ISCs and that C57BL/6 WT mice are not limited by mosaic expression patterns and have ISC markers similar to those of the human intestine, we isolated crypts from both species of mice. As the results showed, ISCs are interspersed between Paneth cells in intestinal crypts (Figure S2A). The isolated crypts initially form villus‐like spherical structures with a closed‐loop hollow lumen, and then the cyst buds up, differentiates into a crypt‐like structure, and finally forms a mature organoid structure (Figure S2B). Lgr5‐GFP^+^ cells were present in the crypt‐like domain of the organoid (Figure S2A). To verify the cell types in C57BL/6 WT mouse‐derived organoids, the cellular markers LGR5, villin, MUC2, lysozyme and chromogranin A (CHGA) were stained to characterize the ISCs, enterocytes, goblet cells, Paneth cells and enteroendocrine cells, respectively (Figure 1B). Our results demonstrated that all of these markers were present in the established intestinal organoids, and granule‐containing Paneth cells could also be clearly observed in the organoid crypts by using an inverted microscope (Figure S2C). These results demonstrated that the established intestinal organoids displayed mature morphology. We subsequently explored the impact of TiO_2_ NPs on ISC homeostasis using the in vitro model and found that the addition of 50 μg/mL TiO_2_ NPs significantly inhibited 3D structure formation and decelerated the budding events of intestinal organoids (Figure 1C–G and Figure S3A). In addition, the presence of TiO_2_ NPs considerably decreased the percentage of ISCs in *Lgr5‐eGFP‐IRES‐creERT2* and C57BL/6 WT mouse‐derived organoids (Figure 1H and Figure S3B,C), demonstrating that TiO_2_ NPs affected the homeostasis of ISCs in the 3D in vitro models. Further investigation revealed that the expression of stemness markers (*Lgr5* and *Oct4*) in C57BL/6 WT mouse‐derived organoids was considerably reduced (Figure S3D). These results thus demonstrated that TiO_2_ NPs could perturb ISC homeostasis.

Since the Wnt, BMP and Notch signalling pathways play central roles in maintaining the homeostasis of ISCs.^33^, ^34^, ^35^, ^36^, ^37^ We subsequently asked whether intracellular TiO_2_ NPs affected the activity of these signalling pathways. Our results showed that TiO_2_ NPs had little effect on the protein levels of Hes family bHLH transcription factor 1 (HES1) and p‐SMAD1/5, which is well‐known Notch signalling target gene and BMP signalling reporter, respectively. However, the expression of the Wnt target gene *c‐Myc* was largely reduced in the presence of TiO_2_ NPs, and the addition of TiO_2_ NPs markedly reduced the protein level of β‐catenin, a key transcriptional activator to be degraded in the inhibition of Wnt signalling (Figure 1I and Figure S4). Further quantitative real‐time PCR (qRT–PCR) analysis confirmed that the incubation of TiO_2_ NPs markedly attenuated the transcriptional outputs of Wnt pathway target genes, including *c‐Myc*, *Axin‐2* and *Egfr*, while it had little effect on the mRNA levels of *Vegf* and *Pten*, which have also been reported to regulate stem cell homeostasis^38^, ^39^ (Figure 1J). These findings suggested that TiO_2_ NPs perturbed ISC homeostasis by repressing Wnt signalling.

### 2.3 Wnt signalling blockade by TiO_2_ NPs impairs primary human ISC homeostasis

**FIGURE 2:**
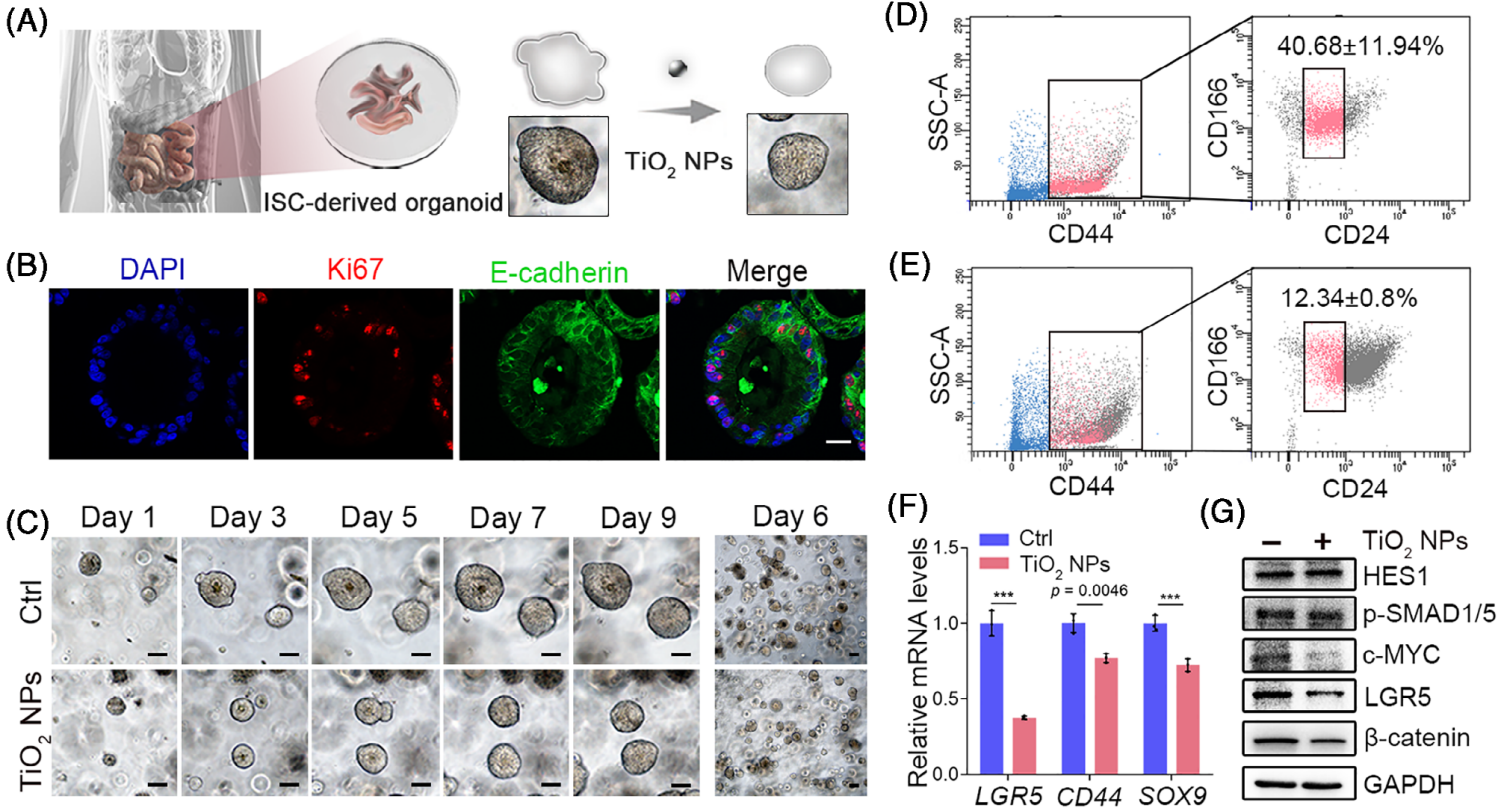
Blockade of Wnt signalling by TiO_2_ NPs disturbs the homeostasis of ISCs in human small intestinal organoids. (A) Schematic representation of the effects of TiO_2_ NPs in human intestinal organoids. (B) Representative images of immunofluorescence staining for E‐cadherin and Ki67 in each group are shown. Scale bar, 20 μm. (C) Time course of an isolated single human small intestinal crypt growth in the absence or presence of TiO_2_ NPs. Scale bars, 50 μm. (D, E) CD44, CD24 and CD166 combination identifies ISCs in human small intestinal organoids in the absence (D) or presence (E) of TiO_2_ NPs. Sequential FACS plots and gates as indicated. (F) qRT–PCR was used to determine the expression of human ISC markers in different groups. (G) The effects of TiO_2_ NPs on the levels of the indicated proteins in human small intestinal organoids. Data are represented as the mean ± SD (*n* = 3). Student's *t*‐test, ***p < 0.001.

Encouraged by the results observed in mouse‐derived 3D models, we established human small intestinal organoids to further validate the effects of TiO_2_ NPs on ISC homeostasis (Figure 2A). The crypts of healthy human small intestine were isolated and cultured as previously described.^40^ In Wnt‐rich culture medium, human small intestinal crypts undergo multiple crypt fission events and finally generate cystic organoids, which comprise mainly stem cells and their highly proliferating progenitor cells. The organoids were stable in culture over passage (Figure S5). Histological analysis showed well‐developed intestinal epithelial structures in human small intestinal organoids expressing proliferative epithelial E‐cadherin (Figure 2B). Our results revealed that the presence of TiO_2_ NPs significantly impaired human ISC homeostasis, as the formation and growth of intestinal organoids were markedly decreased (Figure 2C). CD44^+^CD24^−/lo^CD166^+^ intestinal epithelial cells were identified as putative human ISCs.^32^ The percentage of ISCs in each group was examined by FACS. Consistent with the results observed in mouse intestinal organoids, the presence of 50 μg/mL TiO_2_ NPs considerably reduced the proportion of ISCs in human organoids (Figure 2D,E). In addition, the transcriptional outputs of *Lgr5*, *CD44* and *SOX9*, which have been identified as markers of ISCs, were also significantly compromised in the presence of TiO_2_ NPs (Figure 2F). Since Wnt signalling plays an important role in maintaining the homeostasis of human ISCs, we examined the protein levels of Wnt target genes. The results showed that the expression of c‐MYC and β‐catenin was largely reduced, while the protein levels of HES1 and p‐SMAD1/5 were not affected (Figure 2G and Figure S6). These results verified that TiO_2_ NPs perturbed the homeostasis of ISCs by suppressing Wnt signalling in human small intestinal organoids.

### 2.4 TiO_2_ NPs trigger non‐canonical endocytic trafficking of the Wnt receptor LRP6

**FIGURE 3:**
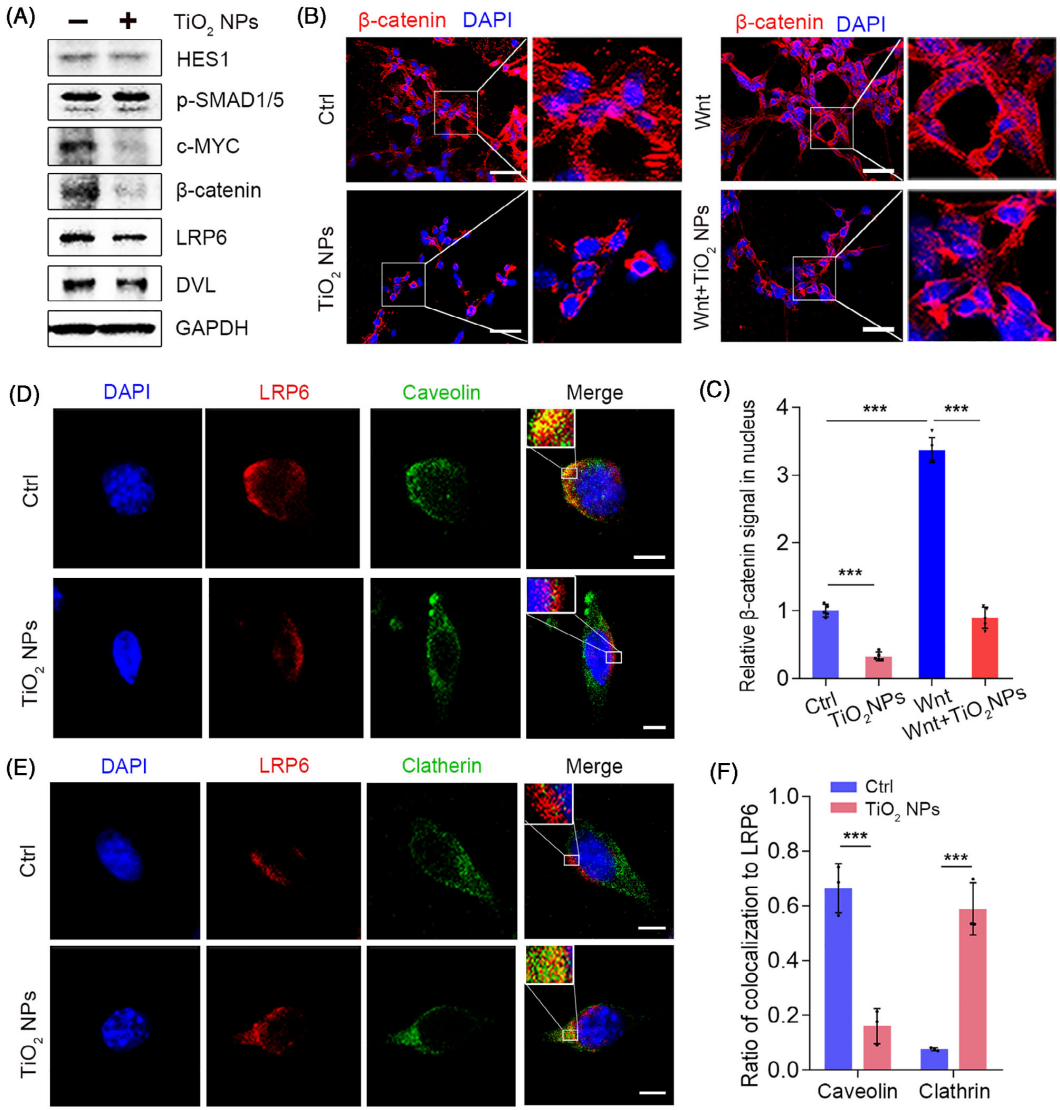
TiO_2_ NPs inhibit Wnt signalling activity by triggering clathrin‐mediated endocytic trafficking of LRP6. (A) The effects of TiO_2_ NPs on the expression of the indicated proteins in CT26 cells. (B) The effects of TiO_2_ NPs on the nuclear entry of β‐catenin in the absence and presence of WNT3A. Scale bars, 50 μm. (C) Quantification of the β‐catenin signal in nucleus with or without TiO_2_ NPs in the absence or presence of WNT3A. Data are represented as the mean ± SD (*n* = 5). Student's *t*‐test, ***p < 0.001. (D) The impact of TiO_2_ NPs on the colocalization of LRP6 with caveolin. Scale bars, 5 μm. (E) The impact of TiO_2_ NPs on the colocalization of LRP6 with clathrin. Scale bars, 5 μm. (F) Quantification of the colocalization of LRP6 with caveolin or clathrin in the absence or presence of TiO_2_ NPs. Data are represented as the mean ± SD (*n* = 3). Student's *t*‐test, ***p < 0.001.

Having found that TiO_2_ NPs could reduce the outputs of Wnt signalling, we further investigated the underlying mechanism in intestinal epithelial cells. In agreement with the results in 3D models, the addition of TiO_2_ NPs specifically repressed the expression of c‐MYC and β‐catenin but had little effect on HES1 and p‐SMAD1/5 in CT26 cells (Figure 3A and Figure S7). In addition, the presence of TiO_2_ NPs prevented the nuclear entry of β‐catenin in the absence or presence of WNT3A protein (Figure 3B,C), confirming that TiO_2_ NPs suppressed Wnt signal transduction. Then, we examined the expression of LRP6 and Dishevelled (DVL), two major components upstream of β‐catenin in Wnt signalling, and found that incubation with TiO_2_ NPs did not alter the protein levels of these two proteins (Figure 3A and Figure S7).

Given that the expression of LRP6 was not affected, we further asked whether the endocytic trafficking of LRP6 was altered. It has been reported that the internalization of LRP6 with caveolin is necessary for Wnt signal transduction.^41^, ^42^, ^43^, ^44^ Consistently, our results showed that internalized LRP6 was primarily colocalized with caveolin rather than clathrin. However, the addition of TiO_2_ NPs selectively sequestered LRP6 from caveolin‐mediated endocytosis to the clathrin‐dependent endocytic route (Figure 3D–F). These results were consistent with our findings that TiO_2_ NPs were mainly internalized via the clathrin‐dependent pathway. Together, these data clarified that TiO_2_ NPs regulated Wnt signalling by triggering non‐canonical endocytosis of LRP6.

### 2.5 TiO_2_ NPs perturb ISC homeostasis and induce modest chronic stress in the mouse small intestine

**FIGURE 4:**
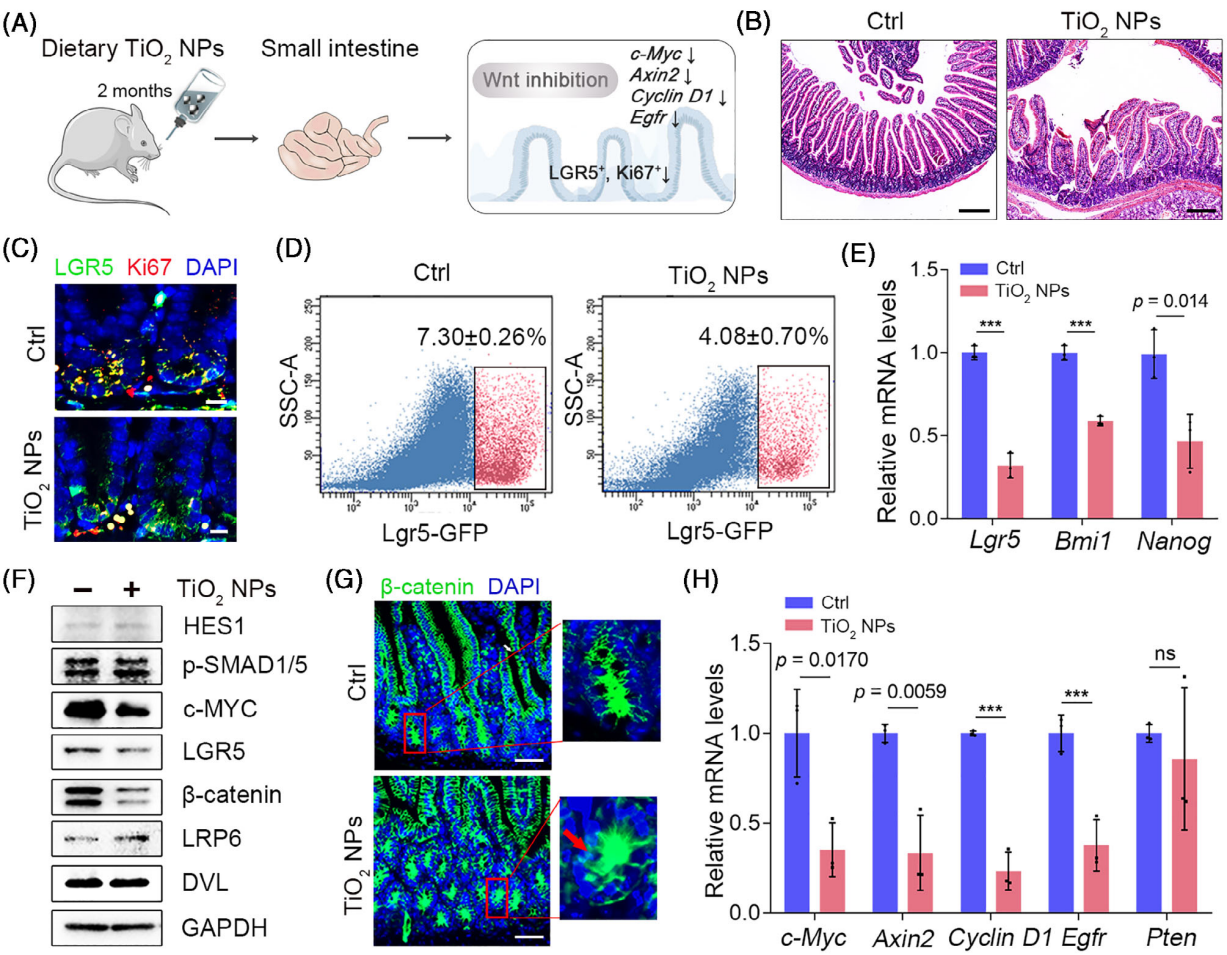
TiO_2_ NPs perturb ISC homeostasis and induce modest stress in healthy intestines in mice. (A) Schematic illustration of the impact of dietary TiO_2_ NPs on ISC homeostasis. (B) Representative images of H&E staining in each group. Scale bars, 200 μm. (C) The colocalization of Lgr5‐GFP and Ki67 in intestinal crypts derived from *Lgr5‐eGFP‐IRES‐creERT2* mice administered TiO_2_ NPs or not. Scale bars, 10 μm. (D) FACS analysis was used to detect Lgr5‐GFP^+^ ISCs in intestinal crypts derived from *Lgr5‐eGFP‐IRES‐creERT2* mice in each group. (E) qRT‐PCR was used to determine the expression of ISC markers in different groups. (F) The effects of TiO_2_ NPs on the levels of the indicated proteins in the intestine of C57BL/6 WT mice. (G) The nuclear entry of β‐catenin in intestine derived from C57BL/6 WT mice in each group was detected. The arrowhead indicates the nuclear entry of β‐catenin. Scale bars, 50 μm. (H) The transcriptional outputs of the indicated genes in each group. Data are represented as the mean ± SD (*n* = 3). Student's *t*‐test, ns means not significant, ***p < 0.001.

Next, we investigated the long‐term effects of dietary TiO_2_ NPs, administered by drinking water, on ISC homeostasis in *Lgr5‐eGFP‐IRES‐creERT2* and C57BL/6 WT mice (Figure 4A). FDA allows 1 wt% TiO_2_ in food, and it was found that more than 36% of particles are nanoscale.^20^ In our study, mice were administered 50 μg/mL TiO_2_ NPs, and the dose was 70 times lower than the FDA‐allowed level. Our results showed that administration of 50 μg/mL TiO_2_ NPs for 2 months had little effect on the length of intestines but induced modest chronic stress in the small intestines, as the epithelial cells of villi were moderately damaged (Figure 4B and Figure S8A,B). In addition, dietary waterborne TiO_2_ NPs significantly hampered the proliferation of ISCs, as the colocalization of Lgr5‐GFP^+^ ISCs and Ki67 was markedly reduced in the small intestine of *Lgr5‐eGFP‐IRES‐creERT2* mice (Figure 4C). Similar results were found in C57BL/6 WT mice (Figure S8C,D). As a result, the proportion of Lgr5‐GFP^+^ cells in *Lgr5‐eGFP‐IRES‐creERT2 mice* and CD44^+^CD24^lo^CD166^+^GRP78lo^/−^ cells in C57BL/6 WT mice was considerably reduced by dietary TiO_2_ NPs (Figure 4D and Figure S9). The mRNA levels of ISC markers such as *Lgr5*, *Bmi1* and *Nanog* were also notably weakened in C57BL/6 WT mice administered TiO_2_ NPs (Figure 4E). Next, we verified the inhibitory effect of TiO_2_ NPs on Wnt signal transduction in vivo. Consistent with the results observed in the 3D in vitro model, dietary TiO_2_ NPs primarily compromised the protein levels of c‐MYC and β‐catenin but had little effect on HES1 or p‐SMAD1/5 (Figure 4F and Figure S10), indicating that TiO_2_ NPs mainly affected Wnt signal transduction in the mouse small intestine. Immunohistological staining of β‐catenin found that the presence of TiO_2_ NPs inhibited β‐catenin nuclear translocation in C57BL/6 WT mouse small intestine (Figure 4G). Further qRT–PCR revealed that TiO_2_ NPs considerably reduced the transcriptional outputs of Wnt signalling, including *c‐Myc*, *Axin2*, *Cyclin D1* and *Egfr*, while they did not alter the mRNA levels of *Pten* and *Vegf* (Figure 4H). These results proved that long‐term dietary TiO_2_ NPs inhibited the proliferation of ISCs by attenuating Wnt signalling and induced modest chronic stress in mouse small intestines.

### 2.6 TiO_2_ NPs suppress radiation enteritis recovery by inhibiting ISC proliferation

**FIGURE 5:**
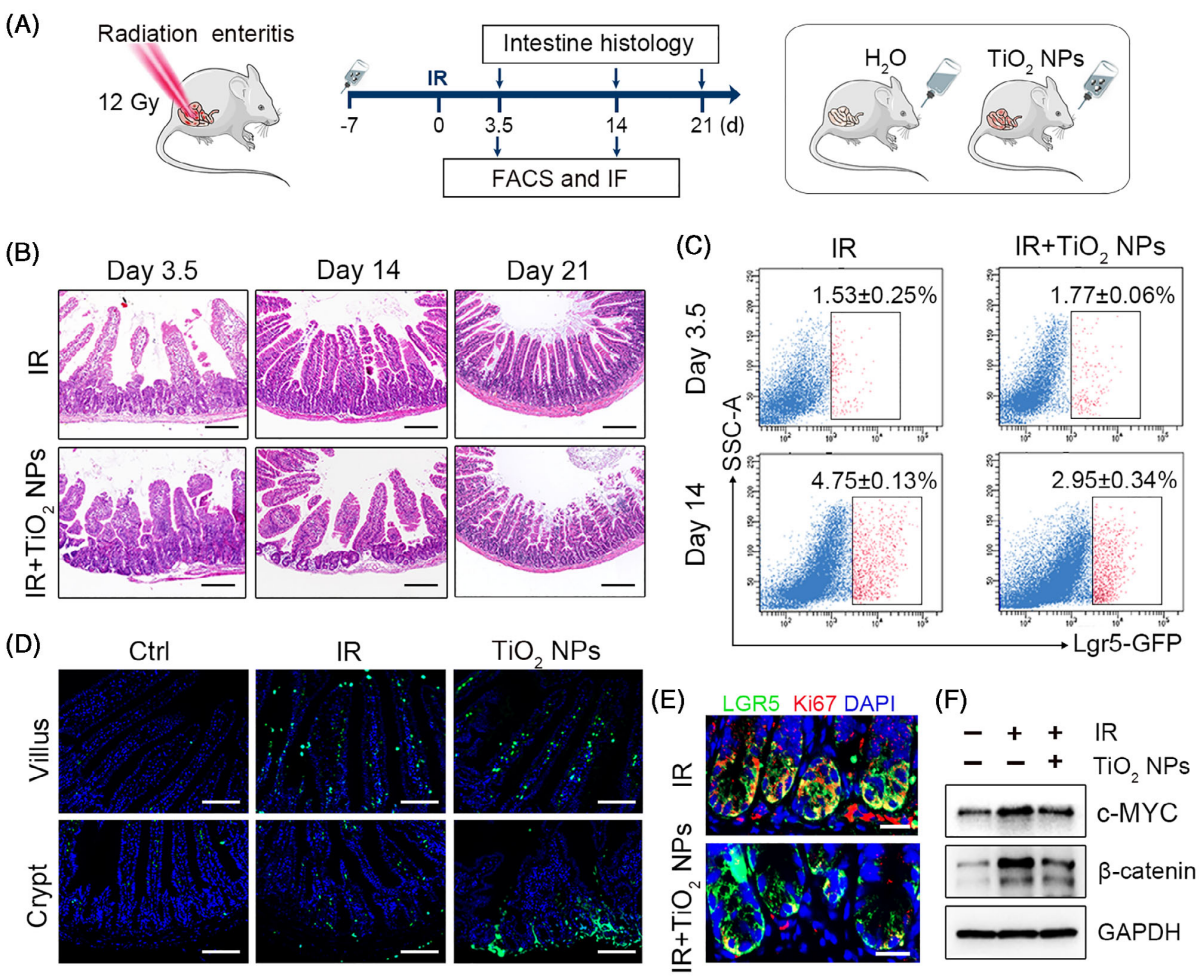
Dietary TiO_2_ NPs hampered the recovery of radiation enteritis by inhibiting the proliferation of ISCs. (A) Schematic illustration of the experimental schedule. (B) Representative images of H&E staining in each group. Scale bars, 200 μm. (C) The percentage of Lgr5‐GFP^+^ ISCs was detected by FACS. Data are represented as the mean ± SD (*n* = 3). (D) Representative TUNEL‐stained images in the villi and crypts of the small intestine on day 14 after radiation in each group. Scale bars, 100 μm. (E) The colocalization of Lgr5‐GFP (green) and Ki67 (red) in the crypts of the small intestine on day 14 after radiation in each group. Scale bars, 20 μm. (F) Western blot analysis of the indicated protein levels in each group.

We further explored the role of TiO_2_ NPs under pathological processes that require a higher threshold of ISC activity to maintain intestinal homeostasis. To address whether dietary TiO_2_ NPs affect the recovery of radiation enteritis, mice were exposed to 50 μg/mL TiO_2_ NPs by drinking water for 4 weeks, with 12 Gy abdominal x‐ray irradiation performed in the second week (Figure 5A and Figure S11A). Our results showed that ionizing radiation (IR) induced visible small intestine oedema and significantly impaired the villi and crypts in the small intestine on day 3.5 after IR (Figure 5B). In addition, the percentage of ISCs was markedly blunted by IR, and dietary TiO_2_ NPs did not aggravate the damage on day 3.5 (Figure 5C and Figure S11B), indicating TiO_2_ NPs did not strengthen IR‐induced enteritis. However, after radiation for 14 days and 21 days, TiO_2_ NP‐treated mice exhibited worse small intestine histology, as the intestinal villus and crypt length were markedly reduced compared to the IR group (Figure 5B and Figure S11C). The number of apoptotic cells in the villi and crypts in TiO_2_ NP‐administered mice was notably higher than that in mice treated with IR alone, indicating that dietary TiO_2_ NPs suppressed the recovery of the small intestinal epithelium (Figure 5D). Given that ISCs are indispensable for small intestine regeneration, we further analysed the impact of TiO_2_ NPs on ISCs in mice after radiation for 14 days. Surprisingly, long‐term intake of waterborne TiO_2_ NPs considerably inhibited the proliferation of ISCs and decreased the proportion of ISCs in radiated mice (Figure 5C,E). Since Wnt signalling was activated to stimulate ISC regeneration after ionizing radiation,^45^ we monitored Wnt signalling activity in radiated mice during this period. The results showed that dietary TiO_2_ NPs markedly prevented the induction of c‐MYC and β‐catenin on day 14 after IR (Figure 5F and Figure S12), suggesting that TiO_2_ NPs inhibited the activation of Wnt signalling during enteritis regeneration. Accordingly, these results demonstrated that chronic uptake of TiO_2_ NPs impeded the recovery of radiation enteritis.

## 3 DISCUSSION

The small intestinal epithelium is a rapidly renewing tissue. The rapid renewal is maintained by ISCs, which are tightly coupled to intestinal health and enteritis.^2^ The imbalance of small ISC homeostasis will compromise the integrity of mucosal barrier and inhibit the recovery of inflammation. In clinic, almost all patients receiving pelvic or abdominal radiotherapy experience radiation enteritis. Recent evidence indicates that the self‐renewal and differentiation of ISCs can be controlled at least in part by diet and nutritional status.^10^, ^11^, ^12^, ^13^, ^14^, ^15^, ^16^, ^17^, ^18^, ^19^ However, studies of food additives in ISC homeostasis are still lacking.

NPs have been widely used in the food industry for their broad benefits, and good biocompatibility of NP is a prerequisite for food‐based applications.^46^, ^47^, ^48^ As one of the most manufactured nanoparticles, TiO_2_ NPs have been commonly used as food additive. However, the impact of chronic NP exposure on ISC homeostasis is still unexplored. Additionally, the doses of TiO_2_ NPs employed in many studies were too high, and TiO_2_ NPs were usually administered to mice by oral gavage, a technique that has been shown to cause stress responses in the gut. Here, we limited the dose of TiO_2_ NPs to 70 times lower than the FDA‐allowed level, and administered TiO_2_ NPs as a part of mouse daily water to minimize the handling stress to assess the impact of TiO_2_ NPs on ISC homeostasis. We found that the presence of low‐dose TiO_2_ NPs significantly hampered primary mouse ISC‐derived organoid formation and growth. In particular, the inhibitory effect of TiO_2_ NPs on ISCs was confirmed in human‐derived intestinal organoids. Mechanistically, TiO_2_ NPs propelled LRP6 from caveolin‐mediated endocytosis to clathrin‐mediated endocytosis to weaken Wnt signalling activity. Further investigation revealed that Wnt signalling blockade by dietary TiO_2_ NPs inhibited ISC proliferation and impeded the recovery of radiation enteritis in vivo. These findings highlight the potential health risk of food‐TiO_2_ NPs on small intestines.

It is worth noting that LGR5 is exclusively expressed in cycling columnar cells (CBCs) at the crypt base.^31^ Just focusing on Lgr5^+^ ISCs exclude some important ISC subpopulations, such as Bmi1^+^ ISCs, which are also important for injury‐induced regeneration.^9^, ^49^, ^50^ Thus, we detected the mRNA level of *Bmi1* and found that dietary TiO_2_ NPs significantly compromised the transcription output of *Bmi1*, indicating that TiO_2_ NPs perturb the homeostasis of different ISC subpopulations. Additionally, LGR5 is not restricted to expression on small intestinal stem cells. In fact, in the colon, hair follicle and stomach, LGR5 also marks stem cells,^31^, ^51^ indicating that TiO_2_ NP‐induced abnormalities in Lgr5^+^ ISCs may also occur in other tissues. In particular, given that LGR5 is also expressed in scattered cells in pre‐malignant mouse adenomas,^52^, ^53^, ^54^ TiO_2_ NPs could potentially be applied to inhibit the progression of diseases in which Lgr5^+^ cells play essential roles.

Taken together, our findings reveal the impact of long‐term TiO_2_ NP exposure on ISC homeostasis and the recovery of radiation enteritis, and disclosed the underlying mechanism. These findings suggest extra precaution during the treatment of radiation enteritis, and provide a better understanding of the relationship between food additives and ISCs.

## 4 METHODS

### 4.1 Mice

*Lgr5‐eGFP‐IRES‐creERT2* mice (Cyagen Biosciences Inc.) and C57BL/6 WT mice (Beijing Vital River Laboratory Animal Technology Co., Ltd.) were used for the experiments. All the animal experiments in this study were approved by the Ethics Committee of the Health Science Center of Xi'an Jiaotong University. Mice were housed in plastic cages and were maintained on a 12‐h light–dark cycle at room temperature (22–26°C) with sterile pellet food and water ad libitum.

### 4.2 Cell culture

The mouse colon cancer cell line CT26 was obtained from the Cell Bank of Chinese Academy of Sciences. The cells were cultured at 37°C in a humidified incubator with 5% CO_2_. RPMI 1640 medium, foetal bovine serum (FBS), streptomycin and penicillin were purchased from Gibco.

### 4.3 Endocytosis of TiO_2_ NPs

CT26 cells were exposed to 50 μg/mL TiO_2_ NPs for 3 h. The cells were carefully rinsed with PBS and fixed with 2% formaldehyde and 2.5% glutaraldehyde for 1 h at 4°C. Cell cultures were cut, and the ultrathin sections were collected and counterstained with tungsten phosphate. The samples were then scanned by TEM. To investigate the endocytosis mechanism of TiO_2_ NPs, CT26 cells were incubated with 400 mM sucrose, 10 μM genistein and 1 μM cytochalasin D for 1 h, which are known as inhibitors of CME, CavMe and macropinocytosis, respectively. After that, the cells were rinsed with PBS three times and incubated with 50 μg/mL TiO_2_ NPs for 3 h. Cells were harvested, fixed and counterstained with tungsten phosphate. The intracellular TiO_2_ NPs were detected by TEM imaging.

### 4.4 Cytotoxicity of TiO_2_ NPs

The cytotoxicity of TiO_2_ NPs in CT26 cells was measured with the Cell Counting Kit‐8 (CCK‐8, Sigma–Aldrich). Briefly, cells were trypsinized and plated in 96‐well plates at a density of 5000 cells/well. Different concentrations of TiO_2_ NPs (10, 50, 100, 150 and 200 μg/mL) diluted in media were added and incubated for the indicated times. After washing with PBS, CCK‐8 solution was added to the cells and incubated for 2 h at 37°C. The absorbance was measured at 450 nm using a microplate reader (Biotek, Cytation 5).

### 4.5 Mouse intestinal crypt isolation and organoid culture

Mouse small intestinal organoids were generated from crypts of *Lgr5‐eGFP‐IRES‐creERT2* and C57BL/6 WT mice. The small intestines were obtained after mice anaesthetised. Flush the intestines with cold PBS by inserting a 5 mL pipette tip into one of the open ends of the intestine. Then, the isolated intestinal tissue was cut into 3‐ to 5‐mm pieces and incubated in 5 mM EDTA for 30 min. The pieces were allowed to settle by gravity, and the supernatant was aspirated. Add 10 mL cold PBS containing 0.1% BSA and suspend tissue pieces up and down three times using a pre‐wetted 10 mL pipette to release crypts. This step was repeated until the supernatant was clear. The fraction was passed through a 70‐μm cell strainer (BD Bioscience) to remove residual villous material. The total fractions were centrifuged at 290 × *g* for 5 min at 4°C. Isolated crypts were counted and pelleted. A total of 1500 crypts were mixed with 50 μL of Matrigel (Corning 356,231) and plated in 24‐well plates. After polymerization of Matrigel, 500 μL of crypt culture medium (Advanced DMEM/F12, 10 mmol/L HEPES, 10 mmol/L GlutaMAX, 100 U/mL penicillin–streptomycin, 1 × N2, 1 × B27, 1 mM N‐acetylcysteine, 500 ng/mL R‐spondin1, 100 ng/mL Noggin, 50 ng/mL EGF and 2.5 μg/mL Primocin) was added. The entire medium was changed every 2 days. For passage, 1 mL GCDR was added and incubated for 10 min at room temperature. Organoids were then mechanically dissociated and transferred to fresh Matrigel. Passage was performed every 5 days with a 1:4 split ratio. The number of viable organoids in triplicate wells was calculated.

### 4.6 Human tissue processing for crypt isolation and organoid culture

Surgically resected small intestinal tissues were obtained from patients at the First Affiliated Hospital of Xi'an Jiaotong University. The Ethics Committee of the Health Science Center of Xi'an Jiaotong University gave approval for this study. All subjects signed the informed consent and the relevant documents were retained. The intestinal tissues were washed and stripped of the underlying muscle layers with surgical scissors. The tissue was cut into 3‐ to 5‐mm pieces and incubated in 2 mM EDTA cold chelation solution (CCS, distilled water with 96.2 mmol/L NaCl, 1.6 mmol/L KCl, 8.0 mmol/L KH_2_PO_4_, 5.6 mmol/L Na_2_HPO_4_, 43.4 mmol/L sucrose, 54.9 mmol/L d‐sorbitol, 0.5 mmol/L dl‐dithiothreitol) for 30 min on ice. After removal of EDTA, the mucosa fragments were resuspended in CCS using a 10‐mL pre‐wetted 10 mL pipette to release crypts. The tissue fragments were allowed to settle down and the supernatant was removed for inspection by inverted microscopy. The resuspension/sedimentation procedure was typically 5–8 times and the supernatants not containing crypts were discarded. The supernatants containing crypts were collected and centrifuged at 200 g for 5 min to separate crypts from single cells. The isolated crypts were embedded in Matrigel and seeded in a prewarmed 24‐well plate (800 crypts in 50 μL Matrigel per well). Matrigel contained 750 ng/mL epidermal growth factor (Peprotech), 1.5 mg/mL Noggin (Peprotech) and 15 mmol/L Jagged‐1 (MCE). The Matrigel was polymerized for 10 min at 37°C, and 500 μL Wnt‐rich culture medium lacking EGF and Noggin (Advanced DMEM/F12, 10 mmol/L HEPES, Glamax, 100 U/mL penicillin–streptomycin, 1 × N2, 1 × B27, 1 mmol/L N‐acetylcysteine, 50% WNT3A conditioned medium, 1000 ng/mL R‐spondin1, 10 mM Nicotinamide, 10 nM gastrin, 500 nM A83‐01, 10 μM SB202190, 10 μM Y‐27632 and 2.5 μg/mL Primocin) was overlaid for the generation of cystic organoids. The entire medium containing 50 ng/mL EGF and 100 ng/mL Noggin was changed every 2 days. Passage was performed every 7 days with a 1:5 split ratio. The number of viable organoids in triplicate wells was calculated.

### 4.7 Histological analysis of organoids

Organoid in Matrigel were resuspended with 10% neutral formalin and incubated for 1 h on ice. After washing with PBS to remove Matrigel, organoids were then fixed overnight at 4°C. Paraffin sections were processed with standard techniques. Haematoxylin and eosin (H&E) and the indicated immunofluorescence staining were performed to characterize the morphology and composition of the organoids. All fluorescence was imaged with a ZEISS Apotome 3.

### 4.8 Statistical analysis

To determine the colony‐forming efficiency, crypts were cultured in 24‐well plate. Seven days after plating, spheres were counted and the colony‐formation efficiency was calculated (number of organoids formed/number of crypts seeded × 100%). The relative organoid area was counted with ImageJ and the budding events in each well were counted to calculate the relative budding events. Student's *t*‐test was used for comparison of two samples with unequal variances.

### 4.9 Western blotting

Samples were lysed and analysed by western blotting to determine the expression levels of the indicated proteins. The following antibodies were used. Mouse anti‐c‐MYC (1:1000), mouse anti‐HES1 (1:1000) and mouse anti‐Dishevelled (1:1000) were from Santa Cruz Biotechnology. Rabbit anti‐p‐SMAD1/5 (1:1000), rabbit anti‐β‐catenin (1:1000) and rabbit anti‐GAPDH (1:1000) were from Cell Signalling Technology. Rabbit anti‐LRP6 (1:1000 for western blot and 1:200 for immunostaining) and mouse anti‐LGR5 (1:1000 for western blot and 1:200 for immunostaining) antibodies were purchased from Immunoway. Mouse anti‐clathrin (1:200 for immunostaining) and mouse anti‐caveolin (1:200 for immunostaining) were purchased from Proteintech.

### 4.10 Confocal imaging

To investigate the endocytic trafficking of the Wnt receptor LRP6, CT26 cells were treated with or without 50 μg/mL TiO_2_ NPs. After 48 h of incubation, the cells were fixed in 4% PFA for 20 min at room temperature, washed with PBS and blocked with goat serum for 1 h. Cells were incubated with the indicated primary antibody at 4°C overnight and the corresponding fluorescent secondary antibody for 2 h at room temperature. After washing, 4′,6‐diamidino‐2‐phenylindole (DAPI) was stained at room temperature for 8 min. All fluorescence was imaged with a confocal laser scanning microscope (ZEISS). The colocalization of clathrin or caveolin with LRP6 was quantified with ImageJ.

### 4.11 Quantitative real‐time PCR

Total RNA from samples was extracted with TRIzol (Ambion) and reverse‐transcribed into cDNA with the ReverTraAce qPCR RT Kit (TOYOBO). SYBR Green Real‐time PCR Master Mix (TOYOBO) was employed to perform quantitative PCR on a Real‐Time PCR System (Bio‐Rad, CFX96). The expression level of the actin or *Gapdh* gene was used as an internal control.

### 4.12 Fluorescence activated cell sorting (FACS) analysis

To investigate the impact of TiO_2_ NPs on the percentage of ISCs, mouse intestines or cultured mouse/human organoids in each group were obtained and mechanically dissociated and centrifuged at 290 g for 5 min to isolate intestinal crypts. Two millilitres of TrypLE Express (Invitrogen), 10 mmol/L Y‐27632 and 0.5 mmol/L N‐acetylcysteine were added and incubated for 30 min at room temperature to dissociate the crypts into single cells. The mixture was passed through a 20‐μm cell strainer to discard the cell clumps and debris, and the remaining cells were centrifuged at 1500 rpm for 5 min at 4°C. Next, the cells were resuspended in staining medium at a concentration of 2 × 10^6^/mL. For C57BL/6 WT mouse‐derived ISC labelling, the following antibodies were used. The Zombie Aqua™ Fixable Viability Kit, PE/Cyanine7 anti‐mouse CD45, APC anti‐mouse CD24 and APC/Cyanine7 anti‐mouse/human CD44 were from Biolegend. Rabbit polyclonal anti‐GRP78 DyLight 488 was purchased from Novus Biologicals. Mouse ALCAM/CD166 PE‐conjugated antibody was purchased from R&D. For human‐derived ISC labelling, cells were stained with PE/Cyanine7 anti‐human CD45, APC/Cyanine7 anti‐mouse/human CD44, APC anti‐human CD24 and PE anti‐human CD166 for 45 min at 4°C. After washing, the cells were analysed by BD FACSCelesta. The cell size gate, based on forward scatter and side scatter, was set up first to exclude cell debris and clumps. A Zombie Aqua™ Fixable Viability Kit was used to discriminate live cells into subpopulations. The other gates were set as shown in figures.

### 4.13 X‐ray radiation treatment

*Lgr5‐eGFP‐IRES‐creERT2* mice and C57BL/6 WT mice (6–8 weeks old) were used for experiments. Mice received abdominal x‐ray irradiation at a dose of 12 Gy using a linear accelerator (Clinac 2100EX, Varian Medical Systems). Mice were sacrificed at 3.5 days, 14 days and 21 days after irradiation to analyse the histology of the small intestine.

### 4.14 Small intestine histology

After the mice were sacrificed, an approximately 3‐cm length of the small intestine was collected, fixed in 4% PFA, dehydrated, embedded in paraffin and sectioned using standard methodologies. Sectioned slides were stained with H&E to observe the impact of TiO_2_ NPs on the morphology of the mouse small intestine in each group. Additionally, rabbit anti‐Ki67 (1:200), rabbit anti‐β‐catenin (1:200) and mouse anti‐LGR5 (1:200) antibodies and a TUNEL Apoptosis Assay Kit were used to stain the sectioned slides to determine the impact of TiO_2_ NPs on ISC homeostasis and radiation‐induced intestinal regeneration. All fluorescence was imaged with a ZEISS Apotome 3.

## AUTHOR CONTRIBUTIONS

Linpei Zhang and Yinli He contributed equally to this work. Xiaojiao Li, Haiyun Song and Yawen Wang conceived the ideas and designed the experiments. Linpei Zhang, Yinli He, Lele Dong, Lin Su, Ruirui Guo, Qinying Luo, Baoyu Gan and Fang Cao conducted the experiments and analysed the data. Xiaojiao Li, Haiyun Song, Yawen Wang and Linpei Zhang wrote the manuscript. All the authors have given approval to the final version of the manuscript.

## CONFLICT OF INTEREST STATEMENT

All authors declare no competing financial interests.

## Supporting information


**Figure S1.** Endocytosis and cytocompatibility of TiO_2_ NPs in CT26 cells.
**Figure S2.** The formation and characterization of mouse intestinal organoids.
**Figure S3.** The impact of TiO_2_ NPs on the homeostasis of ISCs in C57BL/6 WT mouse‐derived intestinal organoids.
**Figure S4.** Full unedited gel for Figure 1I.
**Figure S5.** Time course of isolated human small intestinal crypt growth.
**Figure S6.** Full unedited gel for Figure 2G.
**Figure S7.** Full unedited gel for Figure 3A.
**Figure S8.** The influence of TiO_2_ NP exposure in the small intestines of C57BL/6 WT mice.
**Figure S9.** The impact of TiO_2_ NPs on the percentage of ISCs in intestinal crypts derived from C57BL/6 WT mice.
**Figure S10.** Full unedited gel for Figure 4F.
**Figure S11.** Dietary TiO_2_ NPs did not strengthen IR‐induced enteritis.
**Figure S12.** Full unedited gel for Figure 5F.

## Data Availability

The data that support the findings of this study are available from the corresponding authors upon reasonable request.

## References

[1] Barker N . Adult intestinal stem cells: critical drivers of epithelial homeostasis and regeneration. Nat Rev Mol Cell Biol. 2014;15(1):19‐33. 24326621 10.1038/nrm3721

[2] Sato T , Clevers H . Growing self‐organizing mini‐guts from a single intestinal stem cell: mechanism and applications. Science. 2013;340(6137):1190‐1194. 23744940 10.1126/science.1234852

[3] Kim CK , Yang VW , Bialkowska AB . The role of intestinal stem cells in epithelial regeneration following radiation‐induced gut injury. Curr Stem Cell Rep. 2017;3(4):320‐332. 29497599 10.1007/s40778-017-0103-7 PMC5818549

[4] van der Flier LG , Clevers H . Stem cells, self‐renewal, and differentiation in the intestinal epithelium. Annu Rev Physiol. 2009;71:241‐260. 18808327 10.1146/annurev.physiol.010908.163145

[5] Simons BD , Clevers H . Stem cell self‐renewal in intestinal crypt. Exp Cell Res. 2011;317(19):2719‐2724. 21787769 10.1016/j.yexcr.2011.07.010

[6] Sato T , Vries RG , Snippert HJ , et al. Single Lgr5 stem cells build crypt‐villus structures in vitro without a mesenchymal niche. Nature. 2009;459(7244):262‐265. 19329995 10.1038/nature07935

[7] Gehart H , Clevers H . Tales from the crypt: new insights into intestinal stem cells. Nat Rev Gastroenterol Hepatol. 2019;16(1):19‐34. 30429586 10.1038/s41575-018-0081-y

[8] Schmitt M , Schewe M , Sacchetti A , et al. Paneth cells respond to inflammation and contribute to tissue regeneration by acquiring stem‐like features through SCF/c‐kit signaling. Cell Rep. 2018;24(9):2312‐2328.e7. 30157426 10.1016/j.celrep.2018.07.085

[9] Yan KS , Chia LA , Li XN , et al. The intestinal stem cell markers Bmi1 and Lgr5 identify two functionally distinct populations. Proc Natl Acad Sci U S A. 2012;109(2):466‐471. 22190486 10.1073/pnas.1118857109 PMC3258636

[10] Yilmaz OH , Katajisto P , Lamming DW , et al. mTORC1 in the Paneth cell niche couples intestinal stem‐cell function to calorie intake. Nature. 2012;486(7404):490‐U487. 22722868 10.1038/nature11163 PMC3387287

[11] Tinkum KL , Stemler KM , White LS , et al. Fasting protects mice from lethal DNA damage by promoting small intestinal epithelial stem cell survival. Proc Natl Acad Sci U S A. 2015;112(51):E7148‐E7154. 26644583 10.1073/pnas.1509249112 PMC4697381

[12] Mihaylova MM , Cheng CW , Cao AQ , et al. Fasting activates fatty acid oxidation to enhance intestinal stem cell function during homeostasis and aging. Cell Stem Cell. 2018;22(5):769‐778.e4. 29727683 10.1016/j.stem.2018.04.001 PMC5940005

[13] Beyaz S , Mana MD , Roper J , et al. High‐fat diet enhances stemness and tumorigenicity of intestinal progenitors. Nature. 2016;531(7592):53‐58. 26935695 10.1038/nature17173 PMC4846772

[14] Mao JM , Hu XM , Xiao Y , et al. Overnutrition stimulates intestinal epithelium proliferation through beta‐catenin signaling in obese mice. Diabetes. 2013;62(11):3736‐3746. 23884889 10.2337/db13-0035 PMC3806619

[15] Cheng CW , Biton M , Haber AL , et al. Ketone body signaling mediates intestinal stem cell homeostasis and adaptation to diet. Cell. 2019;178(5):1115‐1131.e15. 31442404 10.1016/j.cell.2019.07.048 PMC6732196

[16] Saito Y , Iwatsuki K , Hanyu H , et al. Effect of essential amino acids on enteroids: methionine deprivation suppresses proliferation and affects differentiation in enteroid stem cells. Biochem Biophys Res Commun. 2017;488(1):171‐176. 28483523 10.1016/j.bbrc.2017.05.029

[17] Peregrina K , Houston M , Daroqui C , Dhima E , Sellers RS , Augenlicht LH . Vitamin D is a determinant of mouse intestinal Lgr5 stem cell functions. Carcinogenesis. 2015;36(1):25‐31. 25344836 10.1093/carcin/bgu221 PMC4303796

[18] Sorrentino G , Perino A , Yildiz E , et al. Bile acids signal via TGR5 to activate intestinal stem cells and epithelial regeneration. Gastroenterology. 2020;159(3):956‐968.e8. 32485177 10.1053/j.gastro.2020.05.067

[19] Wang D , Li P , Odle J , et al. Modulation of intestinal stem cell homeostasis by nutrients: a novel therapeutic option for intestinal diseases. Nutr Res Rev. 2022;35(1):150‐158. 34100341 10.1017/S0954422421000172

[20] Weir A , Westerhoff P , Fabricius L , Hristovski K , von Goetz N . Titanium dioxide nanoparticles in food and personal care products. Environ Sci Technol. 2012;46(4):2242‐2250. 22260395 10.1021/es204168d PMC3288463

[21] Chen X , Mao SS . Titanium dioxide nanomaterials: synthesis, properties, modifications, and applications. Chem Rev. 2007;107(7):2891‐2959. 17590053 10.1021/cr0500535

[22] Lun Pang C , Lindsay R , Thornton G . Chemical reactions on rutile TiO_2_(110). Chem Soc Rev. 2008;37(10):2328‐2353. 18818830 10.1039/b719085a

[23] Chawengkijwanich C , Hayata Y . Development of TiO_2_ powder‐coated food packaging film and its ability to inactivate *Escherichia coli* in vitro and in actual tests. Int J Food Microbiol. 2008;123(3):288‐292. 18262298 10.1016/j.ijfoodmicro.2007.12.017

[24] Cao X , Han Y , Gu M , et al. Foodborne titanium dioxide nanoparticles induce stronger adverse effects in obese mice than non‐obese mice: gut microbiota dysbiosis, colonic inflammation, and proteome alterations. Small. 2020;16(36):e2001858. 32519440 10.1002/smll.202001858

[25] Zhang YL , Duan SM , Liu Y , Wang Y . The combined effect of food additive titanium dioxide and lipopolysaccharide on mouse intestinal barrier function after chronic exposure of titanium dioxide‐contained feedstuffs. Part Fibre Toxicol. 2021;18(1):8. 33596948 10.1186/s12989-021-00399-x PMC7887831

[26] Li X , Song L , Hu X , et al. Inhibition of epithelial‐mesenchymal transition and tissue regeneration by waterborne titanium dioxide nanoparticles. ACS Appl Mater Interfaces. 2018;10(4):3449‐3458. 29318884 10.1021/acsami.7b18986

[27] Andreyev J . Gastrointestinal symptoms after pelvic radiotherapy: a new understanding to improve management of symptomatic patients. Lancet Oncol. 2007;8(11):1007‐1017. 17976611 10.1016/S1470-2045(07)70341-8

[28] Andreyev HJ , Benton BE , Lalji A , et al. Algorithm‐based management of patients with gastrointestinal symptoms in patients after pelvic radiation treatment (ORBIT): a randomised controlled trial. Lancet. 2013;382(9910):2084‐2092. 24067488 10.1016/S0140-6736(13)61648-7

[29] Metcalfe C , Kljavin NM , Ybarra R , de Sauvage FJ . Lgr5+ stem cells are indispensable for radiation‐induced intestinal regeneration. Cell Stem Cell. 2014;14(2):149‐159. 24332836 10.1016/j.stem.2013.11.008

[30] Thurn KT , Arora H , Paunesku T , et al. Endocytosis of titanium dioxide nanoparticles in prostate cancer PC‐3M cells. Nanomedicine. 2011;7(2):123‐130. 20887814 10.1016/j.nano.2010.09.004 PMC3062699

[31] Barker N , van Es JH , Kuipers J , et al. Identification of stem cells in small intestine and colon by marker gene Lgr5. Nature. 2007;449(7165):1003‐1007. 17934449 10.1038/nature06196

[32] Wang F , Scoville D , He XC , et al. Isolation and characterization of intestinal stem cells based on surface marker combinations and colony‐formation assay. Gastroenterology. 2013;145(2):383‐395.e1–21. 23644405 10.1053/j.gastro.2013.04.050 PMC3781924

[33] Scoville DH , Sato T , He XC , Li L . Current view: intestinal stem cells and signaling. Gastroenterology. 2008;134(3):849‐864. 18325394 10.1053/j.gastro.2008.01.079

[34] Takahashi T , Shiraishi A . Stem cell signaling pathways in the small intestine. Int J Mol Sci. 2020;21(6):2032. 10.3390/ijms21062032 PMC7139586 32188141

[35] Farin HF , Van Es JH , Clevers H . Redundant sources of Wnt regulate intestinal stem cells and promote formation of Paneth cells. Gastroenterology. 2012;143(6):1518‐1529 e1517. 22922422 10.1053/j.gastro.2012.08.031

[36] Perochon J , Carroll LR , Cordero JB . Wnt signalling in intestinal stem cells: lessons from mice and flies. Genes (Basel). 2018;9(3):138. 29498662 10.3390/genes9030138 PMC5867859

[37] Pinto D , Gregorieff A , Begthel H , Clevers H . Canonical Wnt signals are essential for homeostasis of the intestinal epithelium. Gene Dev. 2003;17(14):1709‐1713. 12865297 10.1101/gad.267103 PMC196179

[38] Marsh V , Winton DJ , Williams GT , et al. Epithelial Pten is dispensable for intestinal homeostasis but suppresses adenoma development and progression after Apc mutation. Nat Genet. 2008;40(12):1436‐1444. 19011632 10.1038/ng.256

[39] Jiang S , Bailey AS , Swain JR , et al. Hematopoietic stem cells contribute to lymphatic endothelium. Blood. 2006;108(11):473a. 19043576 10.1371/journal.pone.0003812 PMC2583952

[40] Sato T , Stange DE , Ferrante M , et al. Long‐term expansion of epithelial organoids from human colon, adenoma, adenocarcinoma, and Barrett's epithelium. Gastroenterology. 2011;141(5):1762‐1772. 21889923 10.1053/j.gastro.2011.07.050

[41] Jiang Y , He X , Howe PH . Disabled‐2 (Dab2) inhibits Wnt/beta‐catenin signalling by binding LRP6 and promoting its internalization through clathrin. EMBO J. 2012;31(10):2336‐2349. 22491013 10.1038/emboj.2012.83 PMC3364753

[42] Yamamoto H , Sakane H , Yamamoto H , Michiue T , Kikuchi A . Wnt3a and Dkk1 regulate distinct internalization pathways of LRP6 to tune the activation of beta‐catenin signaling. Dev Cell. 2008;15(1):37‐48. 18606139 10.1016/j.devcel.2008.04.015

[43] Yamamoto H , Komekado H , Kikuchi A . Caveolin is necessary for Wnt‐3a‐dependent internalization of LRP6 and accumulation of beta‐catenin. Dev Cell. 2006;11(2):213‐223. 16890161 10.1016/j.devcel.2006.07.003

[44] Liu C , Li P , Chen N , et al. Titanium dioxide nanoparticles trigger non‐canonical receptor endocytosis to inhibit Wnt signaling. J Biomed Nanotechnol. 2017;13(11):1522‐1532. 31271138 10.1166/jbn.2017.2458

[45] Wang S , Han Y , Zhang J , et al. Me6TREN targets beta‐catenin signaling to stimulate intestinal stem cell regeneration after radiation. Theranostics. 2020;10(22):10171‐10185. 32929341 10.7150/thno.46415 PMC7481405

[46] Ji X , Li Q , Song H , Fan C . Protein‐mimicking nanoparticles in biosystems. Adv Mater. 2022;34(37):e2201562. 35576606 10.1002/adma.202201562

[47] Chen N , Wang H , Huang Q , et al. Long‐term effects of nanoparticles on nutrition and metabolism. Small. 2014;10(18):3603‐3611. 24832525 10.1002/smll.201303635

[48] Ji X , Zhou Y , Li Q , Song H , Fan C . Protein‐mimicking nanoparticles for a cellular regulation of homeostasis. ACS Appl Mater Interfaces. 2021;13(27):31331‐31336. 34227383 10.1021/acsami.1c09281

[49] Tian H , Biehs B , Warming S , et al. A reserve stem cell population in small intestine renders Lgr5‐positive cells dispensable. Nature. 2011;478(7368):255‐259. 21927002 10.1038/nature10408 PMC4251967

[50] Sangiorgi E , Capecchi MR . Bmi1 is expressed in vivo in intestinal stem cells. Nat Genet. 2008;40(7):915‐920. 18536716 10.1038/ng.165 PMC2906135

[51] Jaks V , Barker N , Kasper M , et al. Lgr5 marks cycling, yet long‐lived, hair follicle stem cells. Nat Genet. 2008;40(11):1291‐1299. 18849992 10.1038/ng.239

[52] McClanahan T , Koseoglu S , Smith K , et al. Identification of overexpression of orphan G protein‐coupled receptor GPR49 in human colon and ovarian primary tumors. Cancer Biol Ther. 2006;5(4):419‐426. 16575208 10.4161/cbt.5.4.2521

[53] Yamamoto Y , Sakamoto M , Fujii G , et al. Overexpression of orphan G‐protein‐coupled receptor, Gpr49, in human hepatocellular carcinomas with beta‐catenin mutations. Hepatology. 2003;37(3):528‐533. 12601349 10.1053/jhep.2003.50029

[54] van de Wetering M , Sancho E , Verweij C , et al. The beta‐catenin/TCF‐4 complex imposes a crypt progenitor phenotype on colorectal cancer cells. Cell. 2002;111(2):241‐250. 12408868 10.1016/s0092-8674(02)01014-0

