# Supplementary material for: Perturbation of intestinal stem cell homeostasis and radiation enteritis recovery via dietary titanium dioxide nanoparticles
Source: Cell Prolif. 2023 Feb 16;56(8):e13427. doi: 10.1111/cpr.13427 (PMC10392070; doi:10.1111/cpr.13427)
Supplement: Supplementary file 1 — Figure S1. Endocytosis and cytocompatibility of TiO2 NPs in CT26 cells. Figure S2. The formation and characterization of mouse intestinal organoids. Figure S3. The impact of TiO2 NPs on the homeostasis of ISCs in C57BL/6 WT mouse‐derived intestinal organoids. Figure S4. Full unedited gel for Figure 1I. Figure S5. Time course of isolated human small intestinal crypt growth. Figure S6. Full unedited gel for Figure 2G. Figure S7. Full unedited gel for Figure 3A. Figure S8. The influence of TiO2 NP exposure in the small intestines of C57BL/6 WT mice. Figure S9. The impact of TiO2 NPs on the percentage of ISCs in intestinal crypts derived from C57BL/6 WT mice. Figure S10. Full unedited gel for Figure 4F. Figure S11. Dietary TiO2 NPs did not strengthen IR‐induced enteritis. Figure S12. Full unedited gel for Figure 5F. [file CPR-56-e13427-s001.docx]

**Perturbation of Intestinal Stem Cell Homeostasis and Radiation Enteritis Recovery via Dietary Titanium Dioxide Nanoparticles**

Linpei Zhang^1,5^, Yinli He^1,5^, Lele Dong^2^, Chang Liu^4^, Lin Su^1^, Ruirui Guo^1^, Qinying Luo^1^, Baoyu Gan^1^, Fang Cao^3^, Yawen Wang^1,*^, Haiyun Song^4,*^, Xiaojiao Li^1,*^

^1^ BioBank, The First Affiliated Hospital of Xi'an Jiaotong University, Shaanxi, 710061, China.

^2^ Department of Pharmacy, The First Affiliated Hospital of Xi'an Jiaotong University, Shaanxi, 710061, China.

^3^ Center for Translational Medicine, The First Affiliated Hospital of Xi'an Jiaotong University, Shaanxi, 710061, China.

^4^ School of Public Health, Shanghai Jiao Tong University School of Medicine, Shanghai 200025, China

^5^ These authors contributed equally to this work.

*Emails: wangyw1269@xjtu.edu.cn

songhaiyun@shsmu.edu.cn

lixiaojiao@xjtu.edu.cn


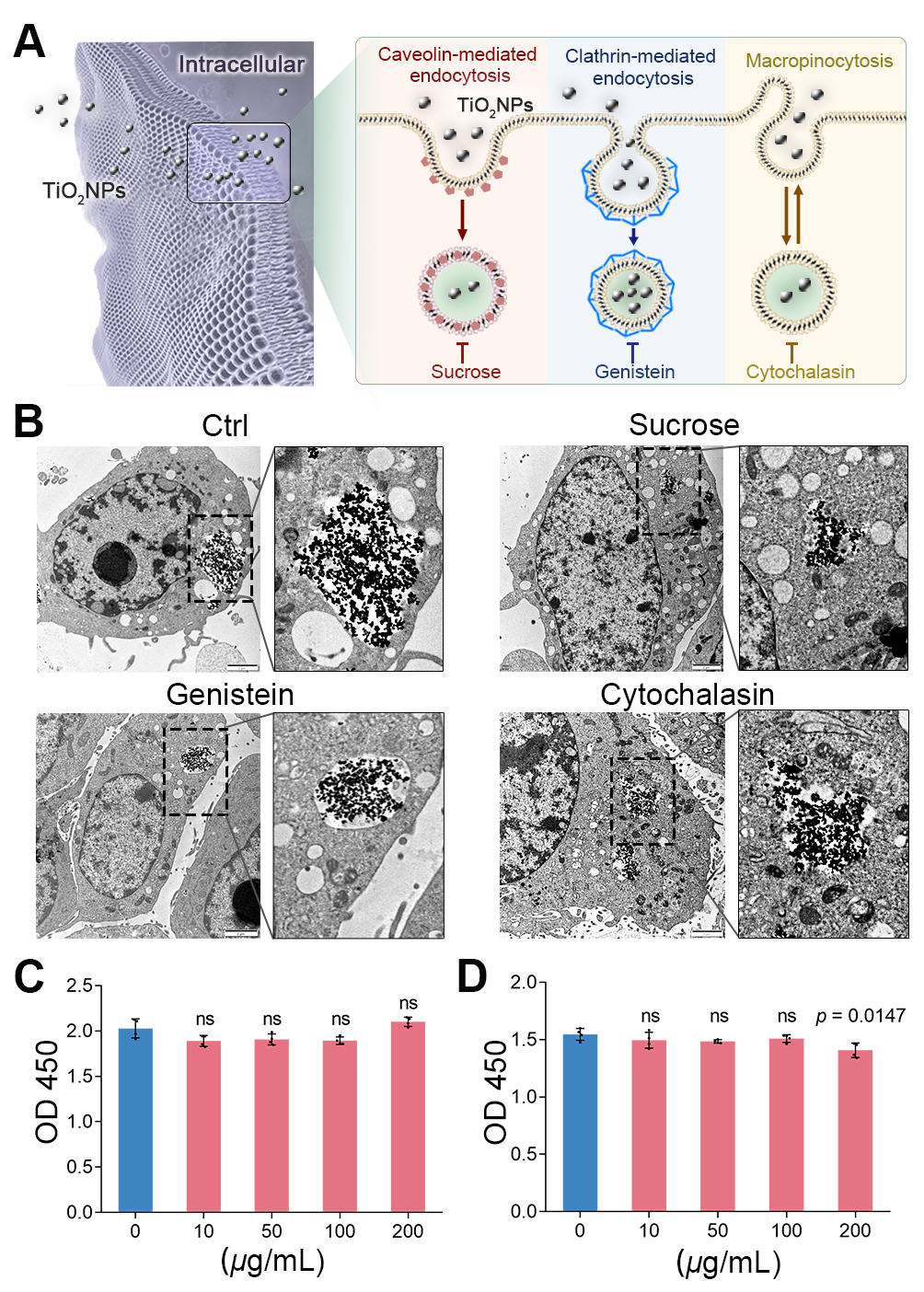


**Supplemental Figure 1. Endocytosis and cytocompatibility of TiO_2_ NPs in CT26 cells. (A)** Three main endocytosis pathways of TiO_2_ NP internalization. **(B)** Effects of endocytosis inhibitors on TiO_2_ NP internalization. Scale bars, 2 *μ*m. **(C)** The cytotoxicity of TiO_2_ NPs at different concentrations (0, 10, 50,100, 150, 200 *μ*g/mL) was measured after 24-hour incubation. **(D)** The cytotoxicity of TiO_2_ NPs at different concentrations was measured after 48-hour incubation. Data are represented as mean ±SD (n=6). Student’s *t*-test, ns means not significant.


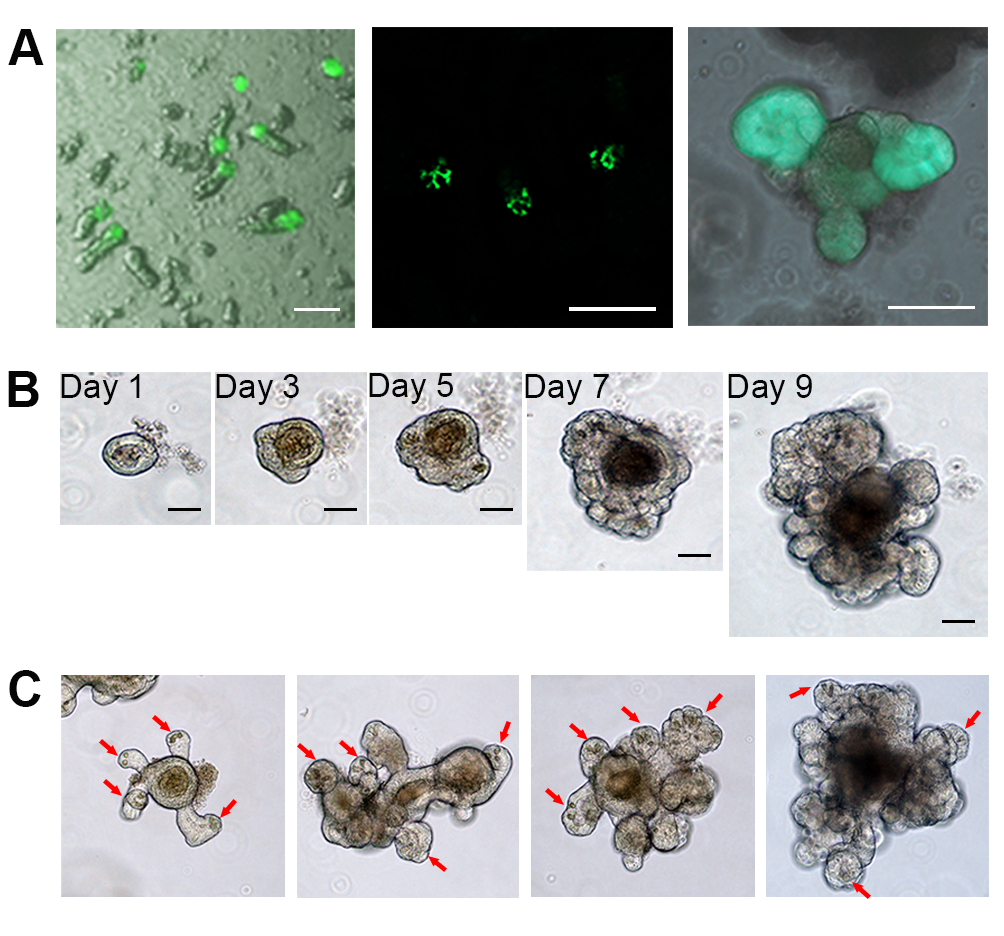


**Supplemental Figure 2. The formation and characterization of mouse intestinal organoids.** **(A)** Crypts isolated from *Lgr5-eGFP-IRES-creERT2* mice efficiently formed large crypt organoids within 7 days. Scale bars, 50 *μ*m. **(B)** Time course of an isolated single crypt growth. Scale bars, 50 *μ*m. **(C)** Differential interference contrast image reveals granule-containing Paneth cells at crypt bottoms (arrows).


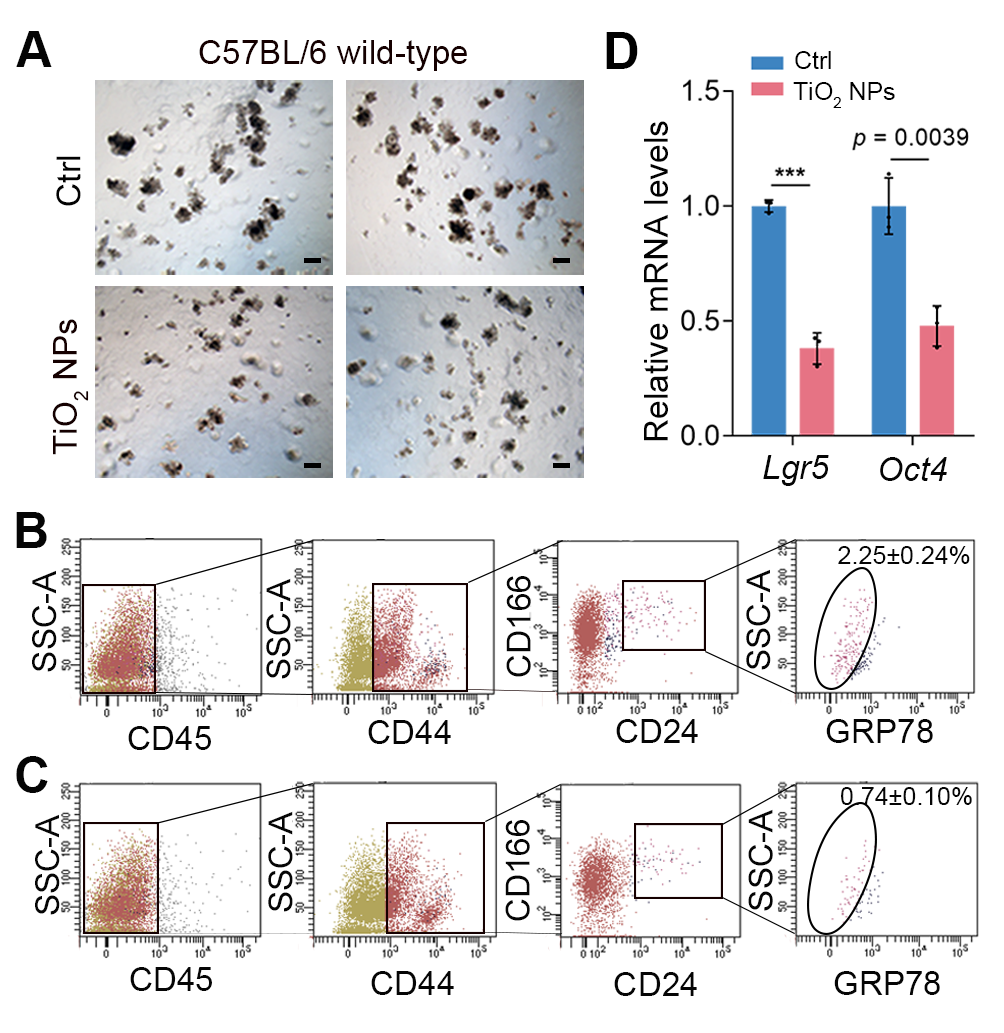


**Supplemental Figure 3. The impact of TiO_2_ NPs on the homeostasis of ISCs in C57BL/6 WT mouse-derived intestinal organoids. (A)** Five days after culturing, TiO_2_ NP exposure compromised the growth of organoids derived from C57BL/6 WT mice. **(B-C)** CD44, CD24, CD166, and GRP78 combination identifies ISCs in intestinal crypts of C57BL/6 WT mice administered with (B) or without (C) TiO_2_ NPs Sequential FACS plots and gates as indicated. **(D)** The relative mRNA levels of stem cell markers were measured by qRT‒PCR. Data are represented as mean ±SD (n=10). Student’s *t*-test, ****p*<0.001.

**
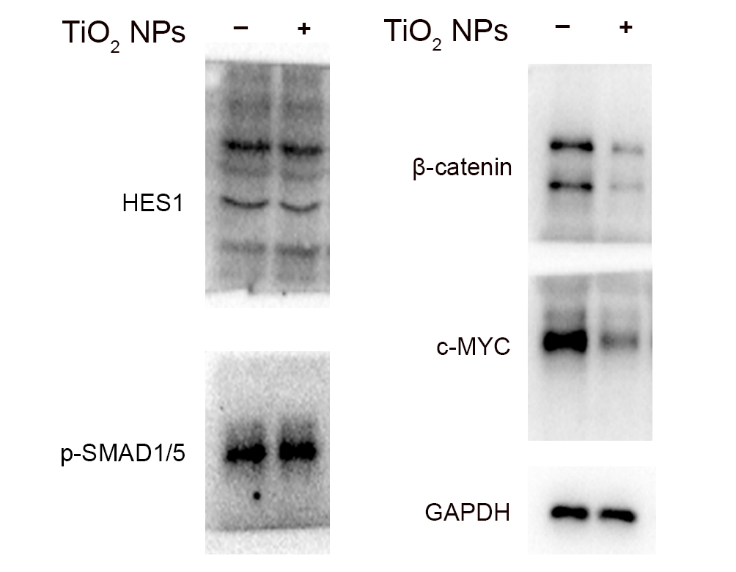
**

**Supplemental Figure 4. Full unedited gel for Figure 1I.**


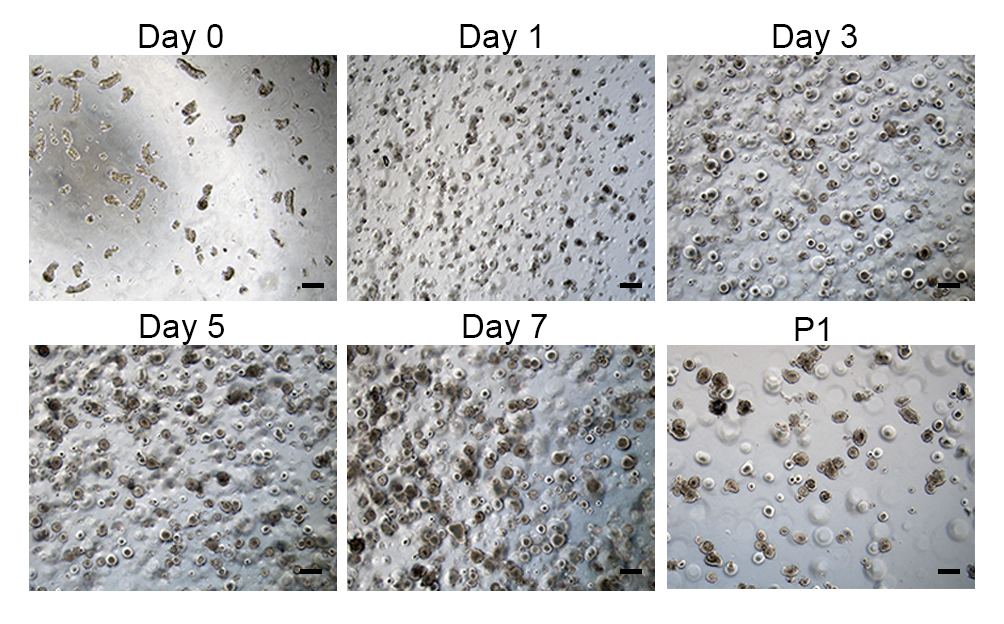


**Supplemental Figure 5. Time course of isolated human small intestinal crypt growth**. Scale bars, 200 *μ*m.


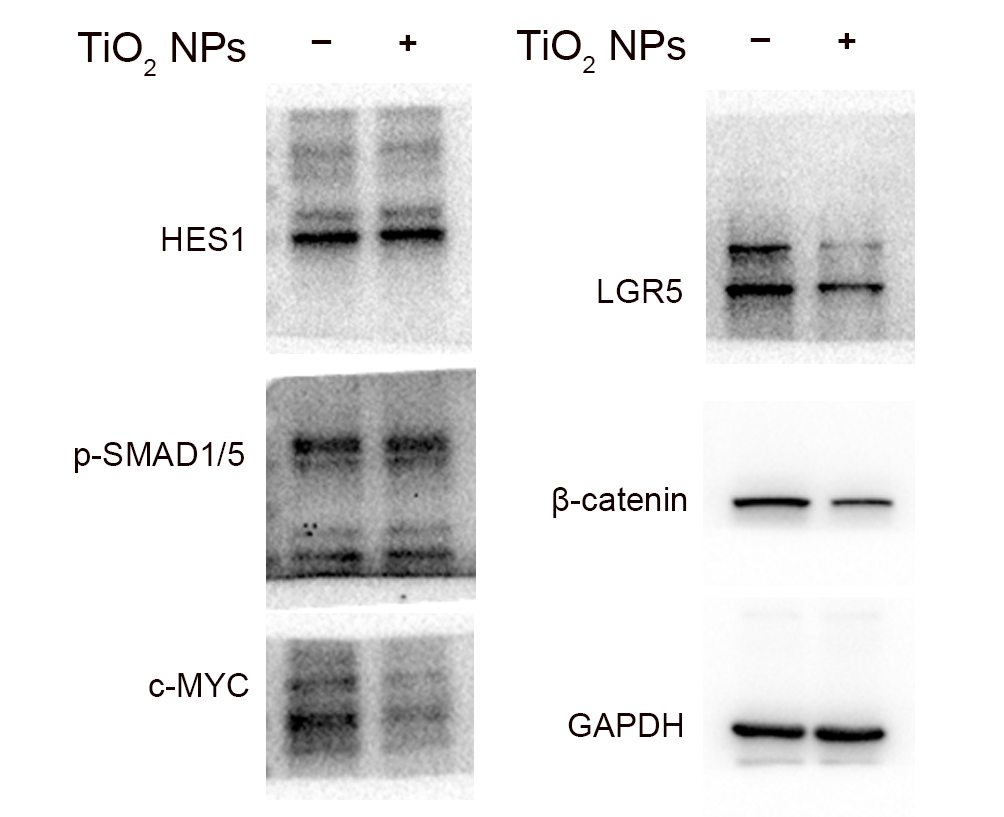


**Supplemental Figure 6. Full unedited gel for Figure 2G.**


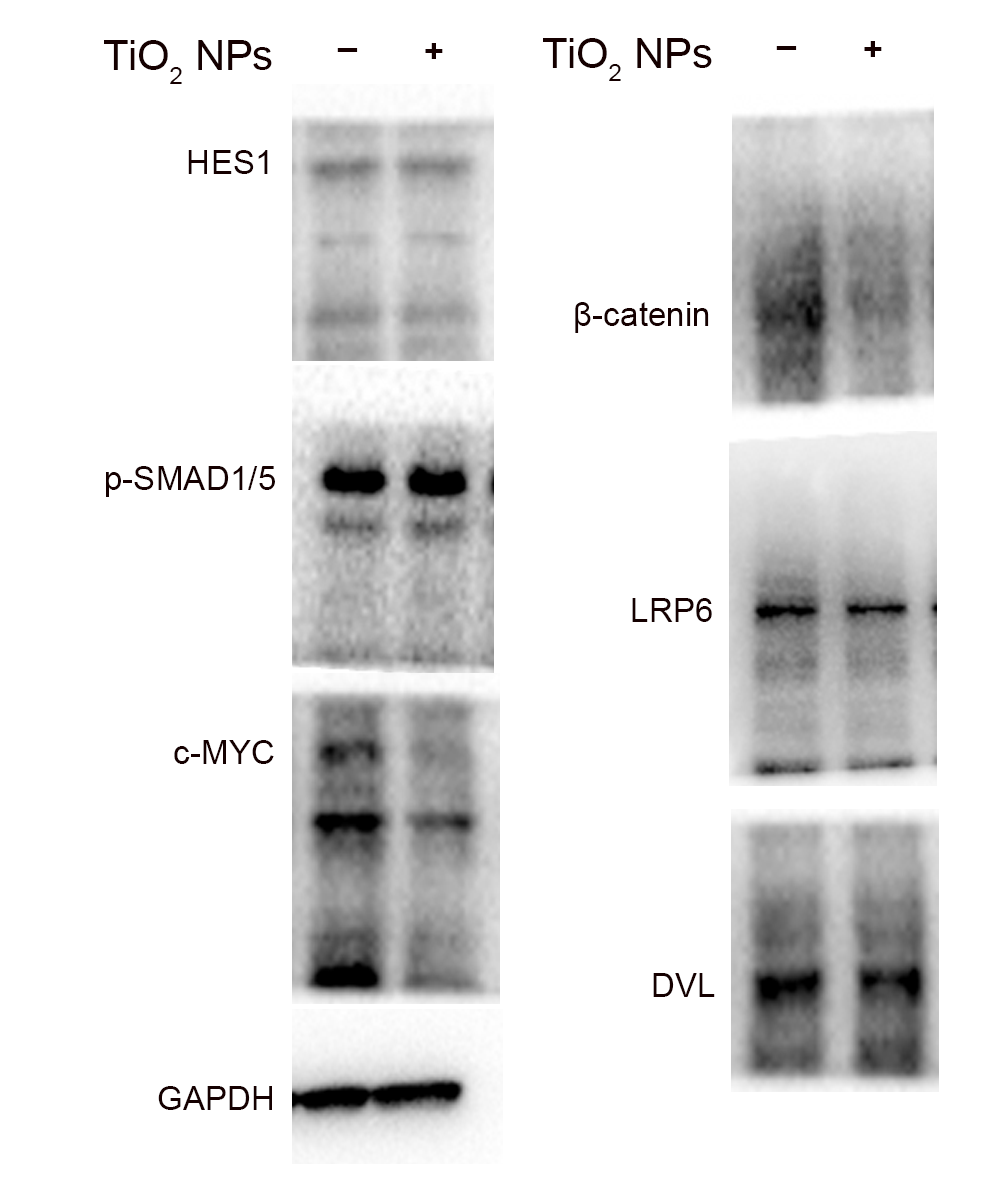


**Supplemental Figure 7. Full unedited gel for Figure 3A.**


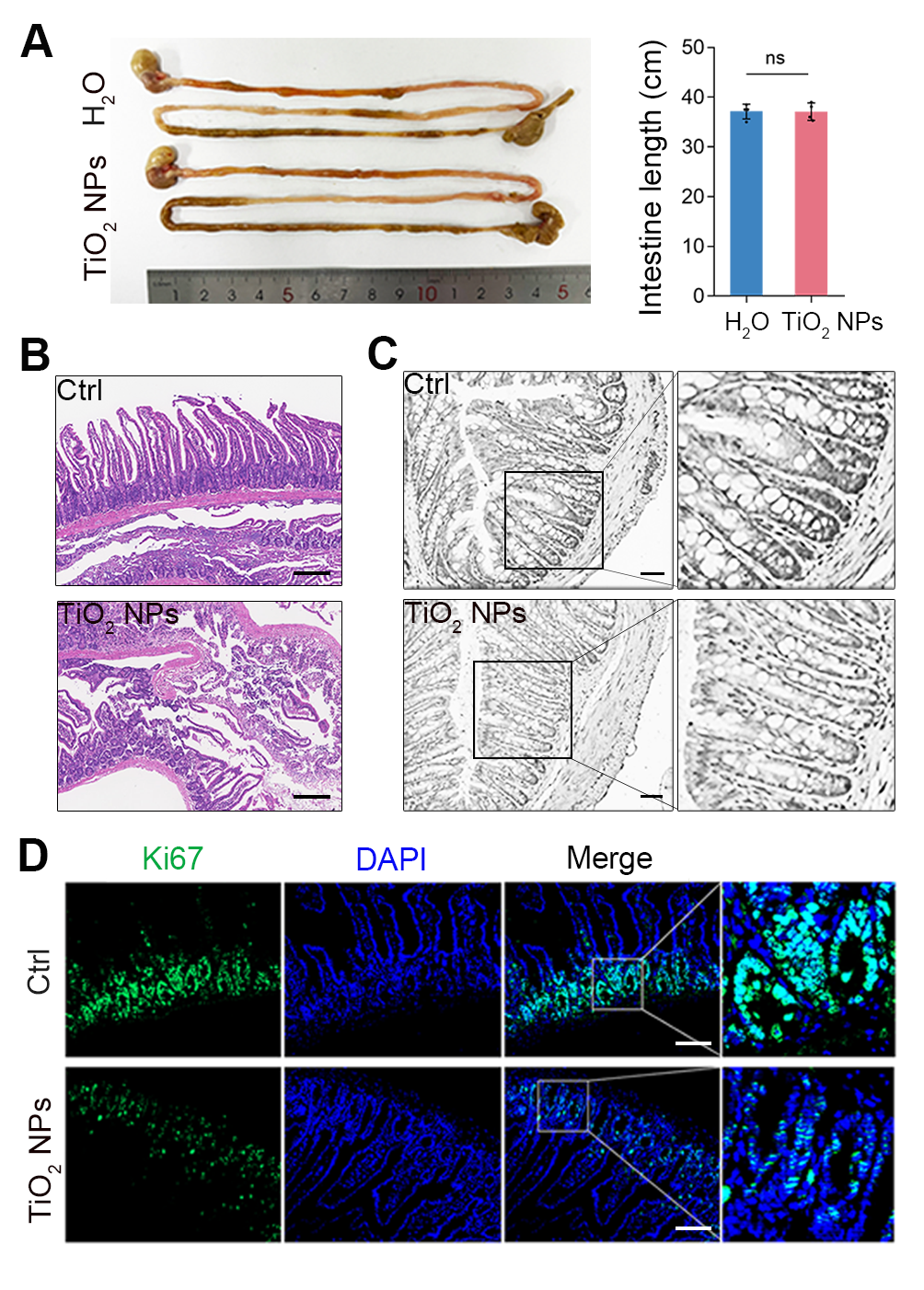


**Supplemental Figure 8. The influence of** **TiO_2_ NP exposure in the small intestines of C57BL/6 WT mice. (A)** Left panel: representative images of mouse intestine at the end of experiment. Right panel: the quantification of the intestinal length in C57BL/6 WT mice in each group. Student’s *t*-test, ns means not significant. **(B)** Representative images of H&E staining in each group. Scale bars, 200 *μ*m. **(C)** Immunohistochemistry of LGR5 in the small intestine from C57BL/6 WT mice in each group. Scale bars, 50 *μ*m. **(D)** Immunofluorescence of Ki67 in each group. Scale bars, 100 *μ*m.


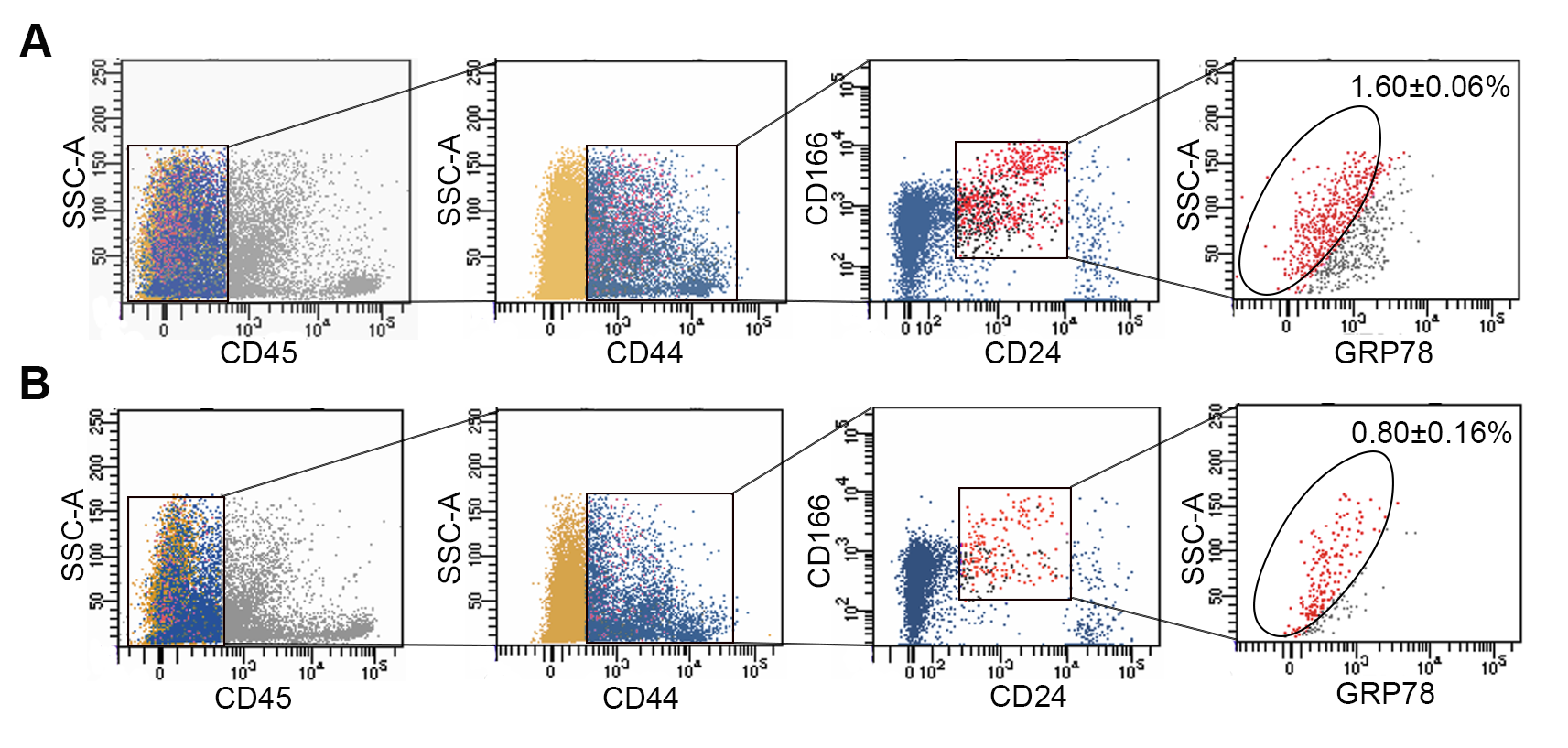


**Supplemental Figure 9. The impact of TiO_2_ NPs on the percentage of ISCs in intestinal crypts derived from C57BL/6 WT mice.** CD44, CD24, CD166, and GRP78 combination identifies ISCs in intestinal crypts derived from C57BL/6 WT mice administered with (A) or without (B) TiO_2_ NPs. Sequential FACS plots and gates as indicated.


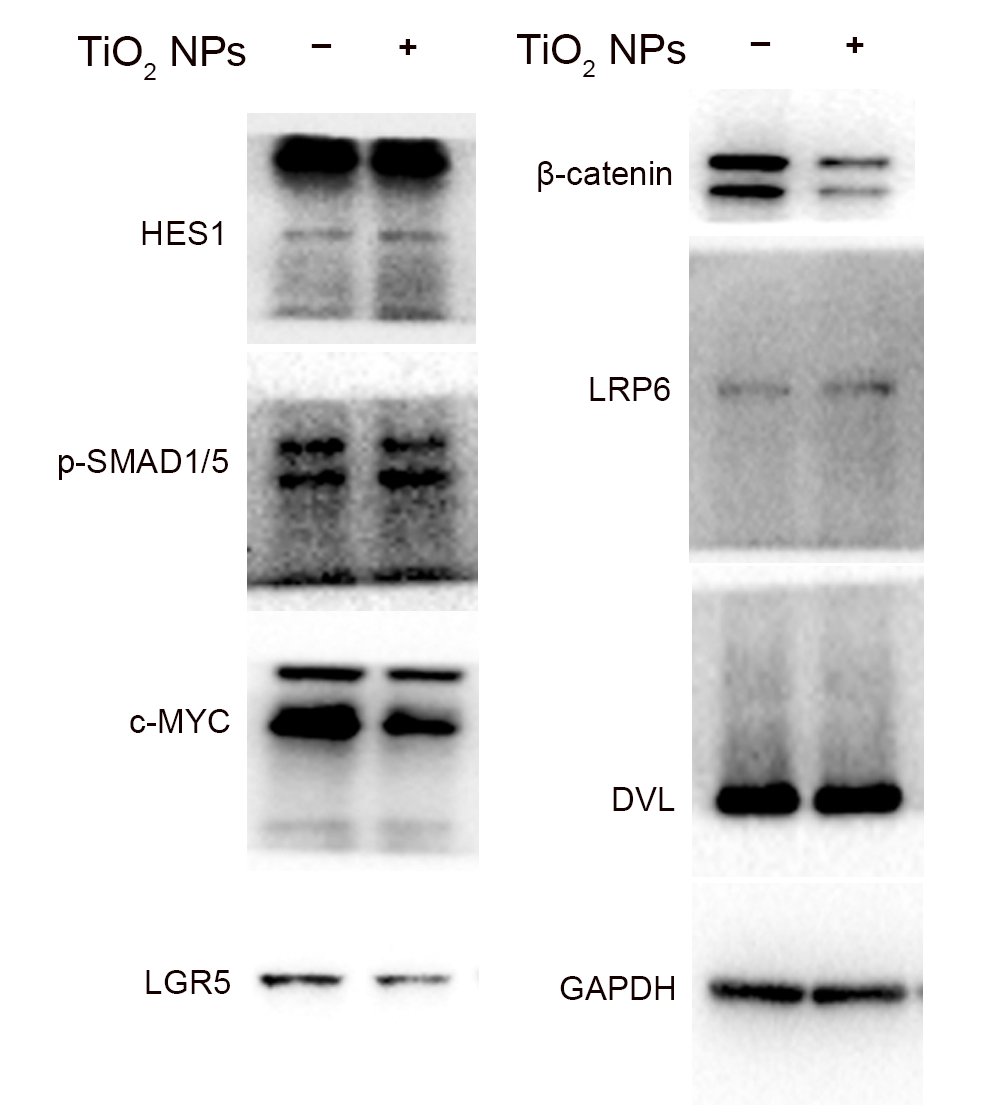


**Supplemental Figure 10. Full unedited gel for Figure 4F.**


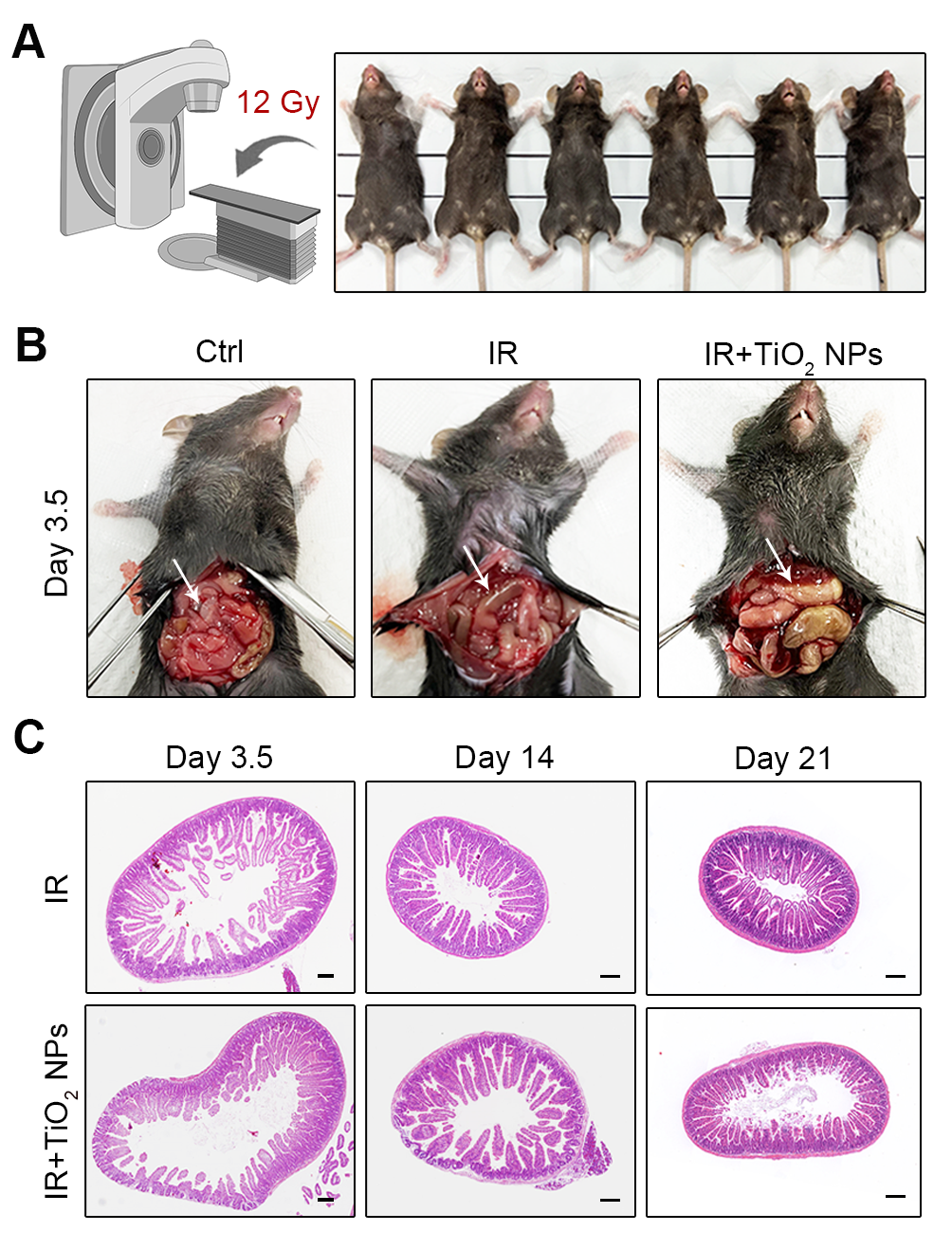


**Supplemental Figure 11. Dietary TiO_2_ NPs did not strengthen IR-induced enteritis. (A)** Mice received abdominal X-ray irradiation at a dose of 12 Gy using a linear accelerator. The linear accelerator created using BioRender.com. **(B)** Ionizing radiation induced visible small intestine edema compared to the control group. **(C)** Representative images of H&E staining in each group. Scale bars, 200 *μ*m.


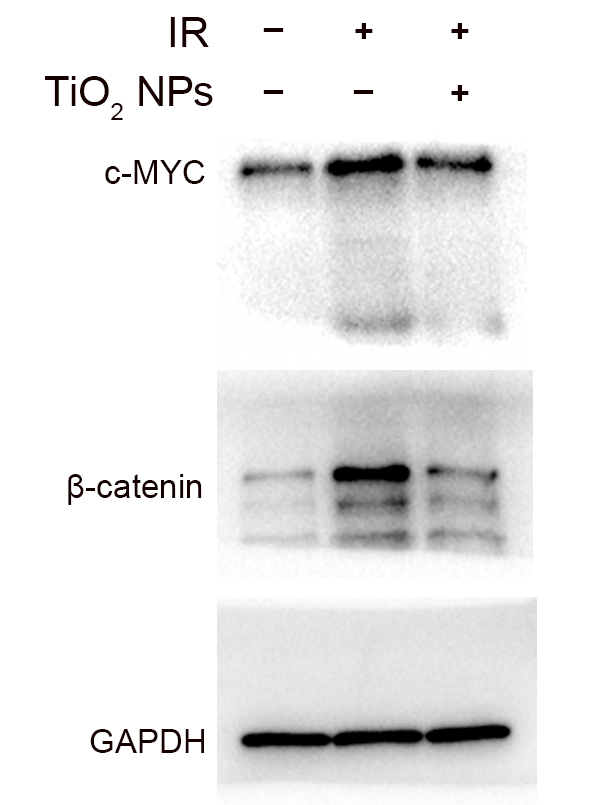


**Supplemental Figure 12. Full unedited gel for Figure 5F.**
